# Multiple independent recombinations led to hermaphroditism in grapevine

**DOI:** 10.1073/pnas.2023548118

**Published:** 2021-04-09

**Authors:** Cheng Zou, Mélanie Massonnet, Andrea Minio, Sagar Patel, Victor Llaca, Avinash Karn, Fred Gouker, Lance Cadle-Davidson, Bruce Reisch, Anne Fennell, Dario Cantu, Qi Sun, Jason P. Londo

**Affiliations:** ^a^Biotechnology Resource Center Bioinformatics Facility, Institute of Biotechnology, Cornell University, Ithaca, NY 14853;; ^b^Department of Viticulture and Enology, University of California, Davis, CA 95616;; ^c^Agronomy, Horticulture and Plant Science Department, South Dakota State University, Brookings, SD 57007;; ^d^Department of Biology, Saint Louis University, St. Louis, MO 63103;; ^e^Department of Biology, Donald Danforth Plant Science Center, St. Louis, MO 63132;; ^f^Corteva Agriscience, Johnston, IA 50131;; ^g^Horticulture Section, School of Integrative Plant Science, Cornell AgriTech, Cornell University, Geneva, NY 14456;; ^h^Grape Genetics Research Unit, US Department of Agriculture–Agricultural Research Service, Geneva, NY 14456

**Keywords:** grapevine domestication, flower sex evolution, recombination, hermaphroditism

## Abstract

Hermaphroditic (perfect) flowers were a key trait in grapevine domestication, enabling a drastic increase in yields due to the efficiency of self-pollination in the domesticated grapevine (*Vitis vinifera* L. ssp. *vinifera*). In contrast, all extant wild *Vitis* species are dioecious, each plant having only male or female flowers. In this study, we identified the male (M) and female (f) haplotypes of the sex-determining region (SDR) in the wild grapevine species *V. cinerea* and confirmed the boundaries of the SDR. We also demonstrated that the SDR and its boundaries are precisely conserved across the *Vitis* genus using shotgun resequencing data of 556 wild and domesticated accessions from North America, East Asia, and Europe. A high linkage disequilibrium was found at the SDR in all wild grape species, while different recombination signatures were observed along the hermaphrodite (H) haplotype of 363 cultivated accessions, revealing two distinct H haplotypes, named H1 and H2. To further examine the H2 haplotype, we sequenced the genome of two grapevine cultivars, 'Riesling' and 'Chardonnay'. By reconstructing the first two H2 haplotypes, we estimated the divergence time between H1 and H2 haplotypes at ∼6 million years ago, which predates the domestication of grapevine (∼8,000 y ago). Our findings emphasize the important role of recombination suppression in maintaining dioecy in wild grape species and lend additional support to the hypothesis that at least two independent recombination events led to the reversion to hermaphroditism in grapevine.

## Significance

We studied the grape sex-determining region (SDR) in 12 *Vitis* genomes and demonstrated its conservation across 556 genotypes including 193 accessions from 47 world-wide wild grapevine species and 363 accessions of cultivated grapevine. Although the grape SDR is recombination free in all wild species, we found two distinct hermaphrodite (H) haplotypes (H1 and H2) among the cultivated grapevines, both chimeras of male (M) and female (f) haplotypes. The two independent recombinations carry different genetic signatures which long predate the domestication of grapevine, suggesting independent evolutions of this trait in wild European grapevine gene pools prior to human domestication.

The evolution of hermaphroditic (perfect) flowers was a key trait in the domestication history of cultivated grapevine (*Vitis vinifera* L. ssp. *vinifera*; hereafter *V. vinifera*) (1, 2), enabling a drastic increase in reliable yields due to the efficiency of self-pollination. In contrast, wild *Vitis* species are dioecious (each plant having only male or female flowers), with known genetic dominance of male (M) > hermaphrodite (H) > female (f) (3, 4). Dioecy is rare in the plant kingdom (5), yet all of the ∼60 extant wild species in the *Vitis* genus are dioecious (6). Given that most ancestors of *Vitis* have hermaphroditic flowers and the rarity of dioecy in flowering plants, this observation suggests a single origin of dioecy in the *Vitis* genus. The *Vitis* genus includes two subgenera: *Muscadinia*, representing only a few species with chromosome number 2n = 40, and *Euvitis*, with chromosome number 2n = 38. Within the *Euvitis*, there are at least 28 wild grapevine species in North America, 30 in Asia, and one in Europe, *V. vinifera* L. *ssp. sylvestris* (hereafter *V. sylvestris*), the wild ancestor of the domesticated species (7). A region spanning ∼150 kb region on chromosome 2 has been identified in multiple genetic studies as the sex-determining region (SDR) in grapevine (4, 8–14). However, due to long divergence time (>40 million years ago, MYA) and high heterozygosity among *Vitis* species, determining the mechanism for the origin of dioecy in the *Vitis* genus and the reversion to hermaphroditism to produce perfect flowers requires further effort.

The primary impediment to understanding the inheritance of flower sex phenotypes and the candidate genes responsible for the various flower sex types in grapevine has been a lack of whole-genome data for wild and cultivated grapevines. Recently, a greater number of cultivated and wild grape genomes have become available (13–17), and the complexity of the flower sex-determining locus in grapevine has begun to clarify. Massonnet et al. (13) examined this region using a comparison of eleven wild and cultivated grape genomes. Structural differences were revealed among M, H, and f haplotypes, delineating candidate genes for both male sterility and female sterility and uncovering a signal of recombination between these candidates in hermaphroditic cultivated varieties. An 8 bp deletion and subsequent frameshift and early termination in the gene *VviINP1* (INAPERTURATE POLLEN1) was implicated as the likely male sterility mutation in *Vitis* spp.; M-linked polymorphisms and expression evidence suggested the transcription factor–coding gene *VviYABBY3* as a female sterility candidate (13). In contrast, Badouin et al. (14) examined this region using a comparative genome approach in *V. sylvestris* and proposed a different candidate for female sterility, *VviAPT3* (14).

While the genetic resolution of the SDR has improved, several key aspects remain unknown. The *Vitis* genus is composed of more than 60 species with distinct gene pools in North America, Europe, and Asia. How conserved is the SDR across these gene pools? Studies of genetic diversity within the wild ancestor and cultivated grape have proposed different hypothetical origins for the reversion to hermaphroditism (2, 12, 13, 18, 19). How conserved is the hermaphroditic haplotype across the diversity of cultivated varieties? Given the contrasting evidence for female sterility genes, can we further clarify the relationships between phenotypes and genotypes? Due to the importance of hermaphroditic flowers in cultivar development, can we develop a comprehensive set of genetic markers to enable the rapid selection of specific flower sex genotypes?

In this study, we answer these questions through the combination of Pacific Biosciences (PacBio) long-read assemblies, whole-genome bulked sequencing, population genetics, transcriptomics, and machine-learning approaches. We document that the genomic boundaries of the SDR are precisely conserved across the entire *Vitis* genus and demonstrate the strong role of recombination suppression in maintaining dioecy in grape. Through our analysis we found the signature of three recombination sites, which combine the wild M and f haplotypes into two distinct H haplotypes, named H1 and H2. In addition, allele-specific transcriptomic analysis including genotypes with either H1 or H2 haplotypes provides support for *VviINP1* and *VviYABBY3* candidate genes for male sterility and female sterility, respectively. Finally, we leverage this knowledge to produce genetic markers across the *Vitis* SDR that predict all possible haplotype combinations from a variety of genomic data types.

## Results

### Whole-Genome Sequencing of Bulked Female and Male Individuals Accurately Define the Boundaries of the Sex-Determining Locus in *V. cinerea*.

**Fig. 1.**
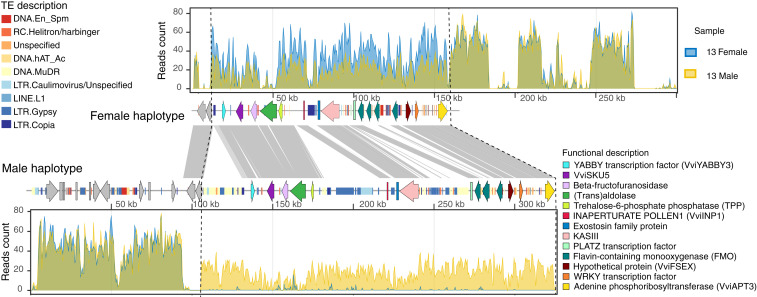
Identification of the two SDRs of *Vitis cinerea* ‘B9’ genome using bulk-sampled whole-genome sequencing. Sequencing depths of uniquely mapped reads from male and female bulked libraries per 500 bp window are plotted along with the two SDR haplotypes. Twofold mapping in female individuals and singlefold mapping in male individuals delineate the SDR from upstream of *VviYABBY3* and to *VviAPT3* (left to right) in both haplotypes. Genes and TEs are illustrated as colored arrows and color-coded blocks, respectively. Sequence similarities between conserved regions are illustrated with gray blocks.

*Vitis cinerea*, a wild North American grapevine species used in both rootstock and scion breeding, was the focus of our initial investigation into the structure of the SDR in grapevine. A hybrid assembly of the male flowering accession ‘B9' using PacBio sequencing reads and Bionano optical maps revealed two scaffolds (Fig. 1 and *SI Appendix*, Fig. S1) with a region similar to the ∼143 kb SDR in the *V. vinifera* ‘PN40024' 12× .v2 reference genome (20). These two scaffolds were 270,484 bp (denoted as haplotype 1) and 325,205 bp (denoted as haplotype 2), respectively. Using shotgun whole-genome sequencing of two pools constructed from 13 male or 13 female accessions of *V. cinerea*, we examined the sequencing read depth differences (*SI Appendix*, Table S1) between the two ‘B9' haplotypes. Sequencing reads uniquely mapped onto these two haplotypes are illustrated in Fig. 1, and the raw read depth was illustrated in *SI Appendix*, Fig. S2. For female (f/f) bulk, twofold read depth was detected, with reads uniquely mapping solely on haplotype 1, whereas for male (M/f) bulk, singlefold sequencing depth mapped to both haplotypes. For additional analysis, we compared these two haplotypes to the SDR of the recently sequenced genome of the female-flowered *V. riparia* ‘Manitoba 37' (16) (f/f) and determined that divergence between *V. cinerea* haplotypes and *V. riparia* ‘Manitoba 37' was significantly smaller for haplotype 1 (two-tailed *t* test, *P* value < 0.007, *SI Appendix*, Fig. S3). Together, these results demonstrate that the *V. cinerea* haplotype 1 corresponds to the f haplotype, while haplotype 2 represents the M form. A twofold coverage in both male and female bulks in the upstream and downstream regions indicate that these regions are not divergent enough to be assembled into two separate haplotypes, delineating the extent of the SDR in *V. cinerea* ‘B9'. The length of f and M haplotypes in *V. cinerea* ‘B9' are 145.5 kb and 226.1 kb, respectively. The size of the f haplotype is very conserved across species, while the M haplotype has a greater variation in size, ranging from 263.4 kb to 805.7 kb in other *Vitis* species (14). In *V. cinerea*, the SDR differentiates into f and M forms beginning roughly at the 5′ untranslated region (UTR) of the plant-specific transcription factor *VviYABBY3* and terminates downstream of the 3′ terminus of *VviAPT3*, an adenine phosphoribosyltransferase gene. The primary source of variation between f and M haplotypes is due to a 24.3% greater transposable element (TE) content in the M haplotype. TE insertions are physically clustered in three regions. The first cluster is found upstream of *VviYABBY3* and is enriched in DNA transposons. The second occurs between *TPP* (Trehalose-6-phosphate phosphatase) and *VviINP1* (INAPERTURATE POLLEN1) and is enriched in LTEs. The final cluster is found between the 3-ketoacyl-acyl carrier protein synthase III gene (*KASIII*) and a PLATZ transcription factor gene and is a mixture of DNA and long-terminal repeat (LTR) transposons (Fig. 1). The genetic distance estimated by the single-nucleotide polymorphism (SNP) density between the f and M haplotypes of *V*. *cinerea* ‘B9' with *V. sylvestris* ‘DVIT3603.16', and *V*. *arizonica* ‘b40-14', did not reveal significant differences. These results show that the structure and boundaries of the SDR in *V. cinerea* is broadly similar to *V. sylvestris* and *V. arizonica* SDRs (13).

### The Sex-Determining Locus Is Conserved Across the *Vitis* Genus.

**Fig. 2.**
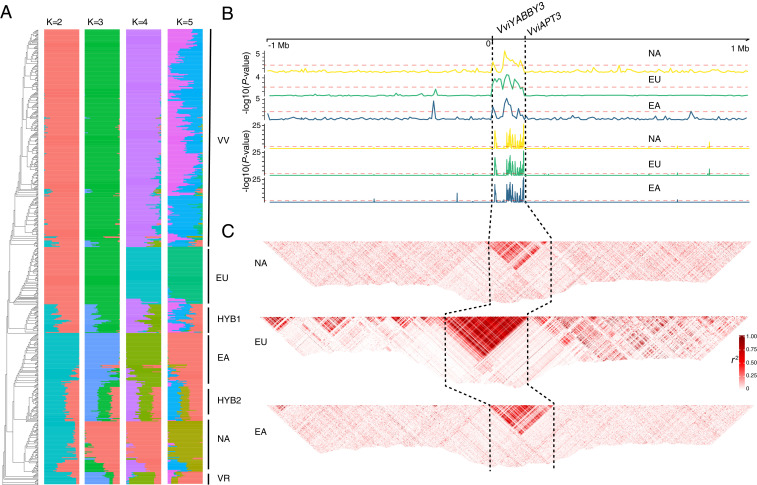
Population characteristics demonstrate the boundaries of the SDR. (*A*) Population structure of 556 grapevine accessions designated as wild North American (NA), wild East Asian (EA), wild European (EU), domesticated grape (VV), hybrid groups (HYB1 and HYB2), and the outgroup *Vitis rotundifolia* (VR). (*B*) Sequence scans of the 2.1Mb region of chromosome 2 hap2 of Cabernet Sauvignon containing the f haplotype. Minor allele frequency (*Upper*) and Hardy–Weinberg equilibrium (*Lower*) for North America (NA), Europe (EU), and East Asia (EA) wild populations. The *x*-axis is the position relative to *VviYABBY3*, and the *y*-axis is the −log10 (*P* value) indicating the significance level of the signal. (*C*) The extent of LD in *r2* detected in wild grape species. Dashed black lines denote the SDR.

To test the conservation of the SDR boundaries across the *Vitis* genus, publicly available whole-genome shotgun sequences were examined for 556 accessions including: 1) 178 wild accessions representing 47 wild grapevine species from North America, East Asia, and Europe, 2) 363 accessions of cultivated grapevine, and 3) 15 accessions of *V. rotundifolia* and other related Vitaceae (Fig. 2 and Dataset S1). To avoid sample size bias between wild and cultivated accessions in the dataset, 36,691 genome-wide ancestry informative markers (AIM) were identified and used to estimate population structure. The muscadine grape, *V. rotundifolia* (2n = 40), was used as the outgroup. The clustering of AIM based on identity-by-state and population structure was conducted with a number of subpopulations (K) ranging from 2 to 5. A value of K = 2 split the dataset into a European gene pool versus a wild North American and East Asian gene pool, likely reflecting the greater overall sampling of cultivated varieties in the dataset. Setting K = 3 split the dataset further by differentiating the North American wild species from European and East Asian gene pools. Individuals were assigned to populations with a probability greater than 0.99 when K = 4 and reflect the four a priori clades in this dataset: a North America clade (NA), an East Asia clade (EA), and two clades representing European grapes, a wild *V. sylvestris* clade (EU), and domesticated *V. vinifera* (VV) (Fig. 2*A*). When K = 5, the domesticated cluster was fractured into two admixed populations, likely reflecting the impact of grape breeding within *V. vinifera* and between wine and table grape types. Two additional clusters of accessions were identified with mixed ancestry between the four core populations and are assumed to be recent hybrid cultivars, denoted as HYB1 and HYB2.

After identifying the population structure in the dataset, several population genetic thresholds were used to infer the extent of the SDR boundaries in the shotgun dataset. As was demonstrated in Massonnet et al. and Badouin et al. (13, 14), the f haplotype appears to contain the least amount of structural variation across the genus, lacking the majority of TEs found in the M and H haplotypes. Given the similarities observed across f SDRs (13, 14) and for ease of comparison with these previous studies, we used the ‘Cabernet Sauvignon' “f” haplotype (12) as the reference sequence. Testing for elevated minor allele frequency, violation of Hardy–Weinberg equilibrium, and patterns of linkage disequilibrium (LD) allowed us to identify 1,066 SNPs that cosegregate with flower sex phenotype (Dataset S2). These metrics demonstrate that the boundaries of the SDR are precisely conserved across the *Vitis* genus (Fig. 2 *B* and *C*).

### Identification of the Critical and Independent Recombination Events Leading to Hermaphroditism.

**Fig. 3.**
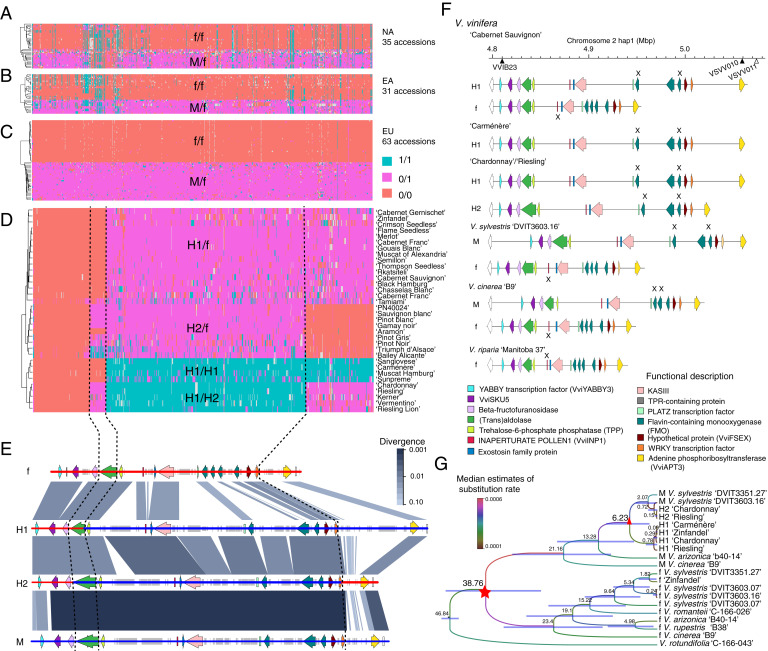
Haplotype structure and recombination events in the SDR of wild and cultivated grapes. (*A*–*D*) Allelic state of the 1,066 sex-linked SNPs in 129 wild and 34 representative cultivated accessions. Colors represent allelic state: red = homozygous reference allele (f allele), magenta = heterozygous, and blue = homozygous alternative allele (M allele). Each row illustrates the genotype of one sample. Abbreviations for gene pool are the following: NA; North American wild, EA; East Asian wild, and EU; European wild. Flower sex genotype is designated as the following: f/f (female flower), M/f (male flower), and H1/f, H2/f, H1/H1, and H1/H2 (hermaphroditic flower). Dashed black lines indicate detected recombination sites. (*E*) Whole-sequence comparison between the f haplotype of *V. sylvestris* 'DVIT3603.16', H1 haplotype of 'Chardonnay', H2 haplotype of 'Chardonnay', and the M haplotype of *V. sylvestris* 'DVIT3603.16'. Chromosomal backbone indicates inferred genetic ancestry: red = f-associated and blue = M-associated. Colored boxes and arrowheads represent TEs and gene models for the region. (*F*) Cartoon model [modified from Massonnet et al. (13)] of structural differences between the various SDR haplotypes observed in sequenced genomes. (*G*) Tree of estimated divergence time of M, f, H1, and H2 haplotypes. The red star indicates the presumed divergence of f and M (and H) haplotypes. The red triangle denotes the divergence of H1 and H2 haplotypes.

In order to determine potential recombination site(s), we examined the genotype of the 1,066 markers that cosegregate with the flower sex phenotype. The entire SDR cosegregated in all wild grapevine species examined (*n* = 129; Fig. 3 *A*–*C*), indicating the absence of recombination in both f and M haplotypes. Large LD blocks encompassing the SDR indicated that recombination has been completely suppressed in wild grapevine species (Fig. 2*C*). LD extent was greater and stronger in the EU clade compared to the NA and EA clades, likely due to a relatively narrow genetic background and a recent origin within the European clade. In contrast, we observed differences of allelic status along with the SDR in all 363 accessions with domesticated *V. vinifera* or mixed germplasm (*SI Appendix*, Fig. S4), highlighting three recombination sites. In Fig. 3*D*, we represent 34 hermaphroditic accessions with four typical combinations of haplotypes.

The most frequently observed recombination event occurs between the transaldolase gene and the *TPP* gene (Fig. 3*E*). Intergenic regions and the first five genes in the SDR, stretching from the upstream region of *VviYABBY3* to the 5′ end of the transaldolase gene, are highly similar to those found in the f haplotype (Fig. 3*E*), while the remaining genes in the locus are highly similar to the M haplotype, suggesting a single crossover event in the SDR at the 5′ end of the transaldolase gene. Numerous clusters of TEs were also observed in this haplotype relative to the f haplotype (*SI Appendix*, Fig. S5). This recombination pattern matches the pattern recently reported by Massonnet et al. in ‘Cabernet Sauvignon', and we designated this H haplotype as H1 (13).

A second recombination pattern was observed for 41 hermaphroditic haplotypes, designated as H2. In the H2 haplotype, recombination occurred in an intron of the transaldolase gene, near the 3′ end of the gene, and colocalized with a TE cluster insertion that increases the predicted gene length relative to the H1 annotation. This TE insertion was also observed in the M haplotypes of the two sequenced male *V. sylvestris* genomes (13), suggesting shared ancestry between the *V. sylvestris* M haplotypes and H2 haplotypes.

The H2 allele is also characterized by an additional recombination site, downstream of the gene encoding a WRKY transcription factor and 47 kb upstream of the annotated *VviAPT3* gene. As a result of these two recombination events, the H2 haplotype has a different pattern of genome similarity across the SDR relative to the H1 haplotype. This pattern of recombination suggests either two independent crossover events within a relatively short distance, or more likely, a double crossover event in the SDR. All biologically possible combinations of the SDR haplotypes were observed in the dataset except H2/H2.

No previously sequenced genome of grapevine includes the H2 haplotype. In order to further examine this haplotype, we sequenced the genome of the cultivar ‘Riesling' (H1/H2) using PacBio sequencing. The high contiguity of the diploid genome (*SI Appendix*, Table S2) allowed us to identify the H1 and H2 haplotypes. These two haplotypes were then used to guide the de novo assembly of the two SDR haplotypes of ‘Chardonnay' (H1/H2) (see *Materials and Methods*). Whole-sequence comparison of ‘Chardonnay' and ‘Riesling' H2 haplotypes with H1 haplotypes showed structural differences between the two forms of H haplotypes, while the structure of H2 haplotypes was found similar to the two *V. sylvestris* M haplotypes (13) (*SI Appendix*, Fig. S6). Gene content in H1 and H2 haplotypes were found to be identical (Fig. 3*C* and *SI Appendix*, Fig. S6).

Divergence time at the SDR was estimated using BEAST software for six wild species from North America (*V. cinerea* [M/f], *V. rupestris* [f/f], and *V. arizonica* [M/f]), East Asia (*V. romanetii* [f/f]), Europe (two *V. sylvestris* [M/f]), and four representative domesticated cultivars: ‘Carménère' (H1/H1), ‘Chardonnay' (H1/H2), ‘Riesling' (H1/H2), and ‘Zinfandel' (H1/f) (*SI Appendix*, Fig. S7). For these estimates we used 1) the conserved region of the locus between *VviINP1* and PLATZ transcription factor genes in the SDR and 2) the *VviYABBY3* portion of the SDR. The divergence time between the outgroup species, *V. rotundifolia*, and the subgenus *Vitis* was constrained at the *Vitis* genus crown age of 46.9 MYA (21). The f haplotype and M haplotype diverged at a mean age of ∼38.6 MYA (95% highest posterior density interval, HPDI, 26.94 to 47.96 MYA), but this divergence overlaps with the crown age of the genus, indicating that f and M haplotypes may have diverged prior to the rise of the *Euvitis* subgenus.

In *V. arizonica* ‘b40-14', six LTRs were estimated to have been inserted in the M haplotype 30 MYA and a H1 specific cluster of LTR dated from 33 to 68 MYA inserted between *KASIII* and the *PLATZ* transcription factor. Due to the shared TE structure, it is clear that both H haplotypes evolved from some form of M haplotype in this region, and we speculate that historical LTR activity played a role in the initiation of SDR evolution. While the two currently sequenced male *V. sylvestris* genomes appear to represent the ancestral gene pool for the H2 haplotype, no current *V. sylvestris* genome has been identified for the H1 haplotype. Resequencing data, however, demonstrates that there are male *V. sylvestris* that are likely associated with the H1 progenitor gene pool.

Divergence between H1 and H2 haplotypes was estimated at 6.1 to 6.7 MYA, soon after the estimated divergence of the European and Asian clades (Fig. 3*G*), suggesting these two gene pools diverged prior to domestication (∼8,000 y ago). Estimating the divergence time using the conserved region around *VviYABBY3* further supports a divergence between H1 and H2 haplotypes that predates the divergence and presumed domestication of *V. vinifera* from the wild ancestor *V. sylvestris* (*SI Appendix*, Fig. S7). The LTR inserted into the transaldolase gene of the two *V. sylvestris* M and *V. vinifera* H2 haplotypes was dated at ∼6 MYA, further suggesting divergence between the H1 and H2 haplotype gene pools occurred before domestication (*SI Appendix*, Fig. S5). Together, this evidence supports the hypothesis that the two recombination patterns observed in the H1 and H2 haplotypes originated from two independent events.

### Pedigree Relationships Support Separate Gene Pools for H1 and H2 Haplotypes.

**Fig. 4.**
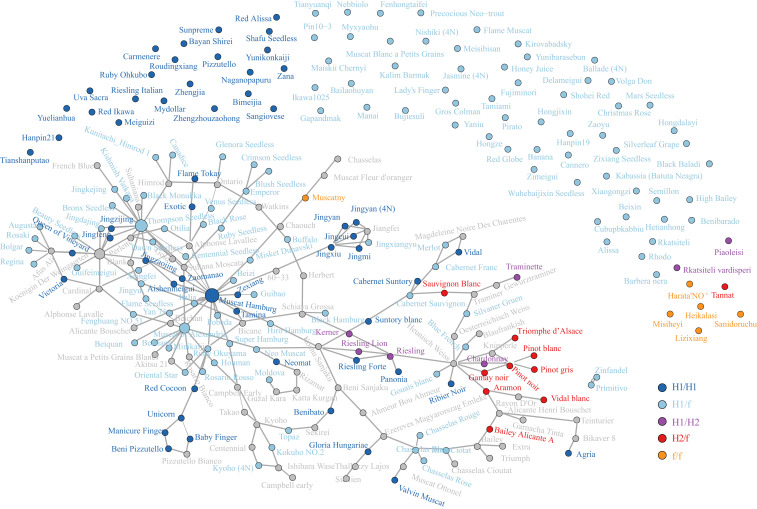
Distribution and pedigree relationships of H1 and H2 haplotypes in sequenced grape cultivars. Lines connecting cultivar dots indicate a pedigree relationship. The size of cultivar dot denotes the frequency of cultivar in the pedigree of sampled cultivars. Unconnected dots lack direct pedigree data. Color of the dot denotes the combination of SDR haplotypes in each genotype. Gray dots are cultivars connecting observed cultivars but are unsampled in genotyping data.

To examine the prevalence of the different H haplotypes among cultivated grapevine accessions, we examined the recorded pedigree relationships for the accessions sampled in this study. Pedigree relationships either through direct parent–offspring association or through unobserved connections could be inferred for 65.1% of the samples (Fig. 4 and Dataset S3). The majority of samples carrying an H haplotype carried the H1 version either in a heterozygous state with the f haplotype (H1/f), with the H2 haplotype (H1/H2), or as homozygous (H1/H1). H1 haplotypes were common in table, raisin, and wine grapes. The H2 haplotype was only observed in a heterozygous state (H2/f or H1/H2) and only in a group of wine grape cultivars of reported provenance to north and west regions of Europe (France and Germany). Tracing of H2 haplotypes through grandparent→parent→child pedigree (e.g., ‘Pinot noir'→‘Knipperelé'→‘Triomphe d’Alsace') indicates the H2 haplotype can be transferred through breeding as well as preserved via clonal propagation.

### Hypothesis Testing Candidate Genes for Sex Determination: *VviINP1*, *VviYABBY3*, and *VviAPT3*.

**Fig. 5.**
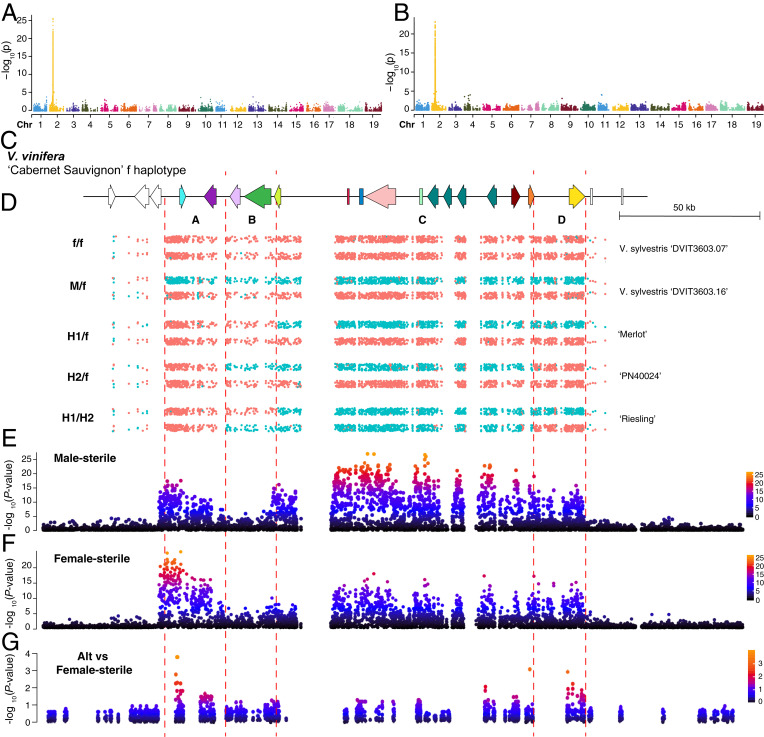
Association between the flower sex phenotype, genotype, and allele-specific expression. (*A* and *B*) Manhattan plots of genome-wide association study (GWAS) for male-sterile (*A*) and female-sterile trait (*B*). (*C*) Gene models for predicted genes located within the sex locus boundary. (*D*) Chromosome painting of male- (blue) and female (pink)-linked SNPs across the SDR. (*E* and *F*) Zoomed Manhattan plot for SDR of GWAS for male-sterile (*E*) and female-sterile traits (*F*). The *y*-axis is an adjusted −log10 *P* value. (*G*) Dot plot demonstrating the association between flower sex phenotype (female-sterile) and alternative-specific gene expression in flower bud tissue. The *y*-axis denotes the −log10 *P* value of the association.

Several different candidate mutations have been proposed for the male sterility and female sterility phenotypes. Most recently, Massonnet et al. identified an 8 bp deletion in the gene *VviINP1* as the likely male sterility mutation and the M allele of *VviYABBY3* as the female sterility gene (13). Badouin et al. in contrast, proposed *VviAPT3* as the female sterility gene (14). Here, we used the pattern of sex-linked SNPs and chromosome painting of these SNPs for the four haplotype combinations H1/f, H2/f, H1/H1, and H1/H2 (all hermaphroditic phenotypes) and allele-specific expression from female, male, and hermaphroditic inflorescences to test these hypotheses (Fig. 5).

Chromosome painting of the sex-linked SNPs partitioned the SDR into four gene regions, A through D (Fig. 5 *C* and *D*). Region A contains two genes, the female sterility gene proposed by Massonnet et al. (13), *VviYABBY3*, and *VviSKU5*. SNPs linked to the male phenotype were significantly associated with this region (Fig. 5*F*). Region B contains two genes encoding beta-fructofuranosidase and transaldolase. No sex-linked SNPs or association with a particular flower sex phenotype were detected in this region. Region C contains eleven candidate genes, and SNPs in this region were significantly associated with the phenotype of male sterility (female). Due to a lack of recombination in this region, the candidate genes cannot be narrowed down to a single specific gene. However, this region includes the current candidate gene for male sterility in grapevine (13), *VviINP1*. Finally, the D region contains a single putative gene, the female sterility candidate gene proposed by Badouin et al. (14), *VviAPT3*. The pattern of sex-linked SNPs among the H haplotype combinations differentiates the role of the four different regions as to their contribution to flower sex. In the A region, all hermaphroditic accessions carry a haplotype that is most similar to that of a female haplotype, while the other three regions can carry a mixture of male- or female-associated SNPs and still retain a hermaphroditic flower (Fig. 5*D*). In the B region, patterns of sex-linked SNPs differ between the H1 and H2 haplotypes due to the two different locations of recombination, with the H1 haplotype maintaining the pattern of female-associated SNPs, while the H2 haplotype carries male-associated SNPs. In the C region, H1/f and H2/f genotypes carry one haplotype with SNPs associated with the male phenotype and one with the female phenotype (heterozygous). H1/H2 genotypes, however, are enriched across the C region for male-associated SNPs. Finally, in the D region, the secondary recombination event observed in H2 haplotypes results in another split pattern by genotype. Here the H1/f and H1/H2 genotypes are heterozygous for sex-linked SNPs while H2/f genotypes are enriched for female-linked SNPs. The presence of the second recombination site in the D region of H2 haplotypes excludes *VviAPT3* as a likely candidate for the female-sterile phenotype. This region can be either M like or f like, yet the flower phenotype is hermaphroditic in either case, leaving evidence supporting region A as the likely female sterility region of the SDR.

To further test the likelihood of *VviYABBY3* as the candidate gene for the female sterility phenotype, we conducted allele-specific transcriptome analysis of 29 accessions representing nine wild species, 13 accessions of domesticated grape, and six bulked female and male samples from three biparental populations with H/H×M/f (*SI Appendix*, Table S3 and Fig. S8). For each polymorphic site, we tested the correlation between the reads that support the reference allele (f haplotype) or alternative allele (M haplotype) with the flower genotype. There was no significant correlation between the f-specific expression with female- or male-sterile phenotype (*SI Appendix*, Fig. S9). The highest correlation was between the M haplotype–specific expression of *VviYABBY3* and the female-sterile phenotype, which indicates potential transcription regulation in determining female sterility (Fig. 5*G*). This result is in agreement with the observation of M-specific transcription factor binding sites observed upstream of *VviYABBY3* (13). The strong association of the A region with the female-sterile trait as well as the occurrence of the second recombination site observed in H2 genotypes indicates that an f-like A region is critical for and found in all currently sampled hermaphroditic samples. The recombination pattern observed in H2 genotypes demonstrates that region D which carries *VviAPT3* is not necessary for the H phenotype, thus excluding this gene as the female sterility candidate. We thus conclude that the female sterility–determining gene is most likely located in the A region, and *VviYABBY3* is the only gene in the A region with a significant signal in genome-wide association study for the female-sterile trait.

### Flower Sex Marker Development and Phenotypic Prediction.

**Fig. 6.**
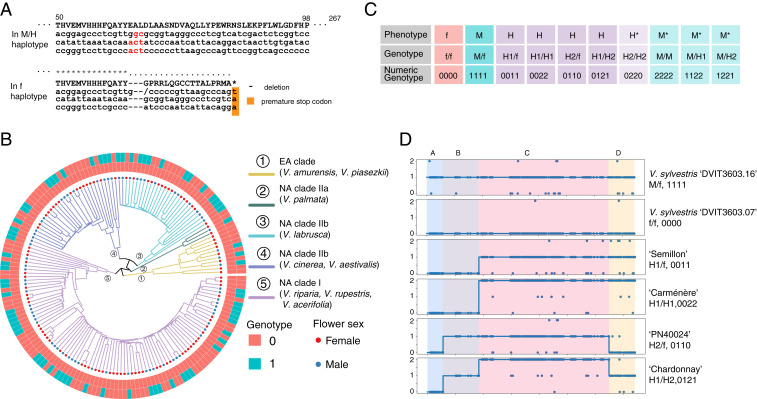
Marker prediction for flower sex in grapevine. (*A*) DNA alignment of *VviINP1* with 8bp indel noted in red for M/H haplotype and missing from f haplotype. (*B*) Flower sex phenotype prediction validation using *VviINP1* deletion and linked C region markers for wild grapevine germplasm (*n* = 167). (*C*) All possible and observed combinations of haplotypes with M, f, H1, and H2 and their flower sex phenotype. Genotypes with an asterisk have not been observed in natural populations. (*D*) Marker prediction haplotyping for flower sex genotypes observed in this study represented as numeric SNP states: 0 = homozygous female allele (f), 1 = heterozygous allele (H/f or M/f), and 2 = homozygous alternative allele (H/H or H/M or M/M).

Our analysis of a marker designed to span the 8 bp deletion in *VviINP1* (14) was accurate in 100% of 167 genotypes representing nine wild grapevine species from North America and East Asia (Fig. 6 *A* and *B* and *SI Appendix*, Table S4) and 187 genotypes from two biparental mapping families, which includes ‘Horizon' (H/H) × *V. cinerea* ‘B9' (M/f) and an F2 family of 16_9_2 (H/f) × 16_9_2 (H/f) (*SI Appendix*, Figs. S10 and S11).

Theoretically, markers that predict male sterility and female sterility separately should distinguish female, male, and hermaphroditic phenotypes when combined. Using this framework, we developed a pipeline to predict the flower sex phenotype based on the genotype of the A region (the female sterility–determining region) and the C region (the male sterility–determining region). Markers in the B and D regions can be used to predict the specific H haplotype. We used Bayes factor hypothesis testing comparing the null hypothesis that all sites are homozygous for the reference sequence and the alternative hypothesis that all sites are heterozygous. Using sites that cosegregated with flower sex, the accuracy of the prediction is 100% based on 193 accessions, comprising 137 wild accessions and 56 cultivars or hybrids with validated phenotypes (Dataset S1). Applying this framework to RNA sequencing data were also successful with an accuracy of 100% for 29 wild accessions and 19 grapevines from cultivars and breeding populations (*SI Appendix*, Table S3).

Due to genetic divergence in the SDR across the *Vitis* genus, prediction power is decreased when surveying the wide variation in species with low marker numbers. Therefore, we used high-density polymorphisms from shotgun sequences to predict the 10 possible genotypes (Fig. 6*C*) that can occur when combining the four SDR haplotypes observed in this study. Using the characteristic reference and alternative allele states for these haplotypes, we computed the numeric genotype that represents the different flower sex haplotypes. Example accessions, their numeric genotype, and corresponding flower phenotype are shown in Fig. 6*D*. Markers we have designed and tested using the amplicon sequencing (Ampseq) and RNase H2-dependent amplicon sequencing (rhAmpSeq) platforms are listed in Dataset S4.

## Discussion

The study presented here documents genomic evidence of multiple independent evolutions of hermaphroditism within domesticated grapevine through exhaustive examination of genetic diversity in the *Vitis* genus. This study tested previous hypotheses about flower sex-determining genes through distribution of sex-linked SNPs, transcriptomics, and recombination patterns, adding support to *VviINP1* and *VviYABBY3* as determining male-sterile and female-sterile phenotypes, respectively. Two unique patterns of recombination between these two candidate loci result in a structurally complemented SDR. The evolution of this important trait from wild grapevine likely enabled early viticulturists to increase the reliability of annual fruit yields and eliminate the need for nearby pollinator vines. Presumably due to ease of vegetative propagation, this hermaphroditic state could be easily selected from the wild and used as breeding stock in the development of the thousands of modern-day domesticated *V. vinifera* cultivars.

### Recombination Signature and Genetic Structure of the SDR in Grapevine.

Rare recombination events shaped the mating system in the *Vitis* genus. The results presented in this study demonstrate that all dioecious, wild grapevines sampled concur with the genetic model of determination in grapevines. Male vines carry both the M and f haplotypes, with M dominant relative to f, while female vines carry only f haplotypes. This strict method of phenotypic expression has fixed sex-associated SNPs across the SDR region. Divergence estimates of the M and f haplotypes overlap with that of the estimates for the divergence of bunch grapes (*Euvitis*, 2n = 38) from muscadine grapes (*V. rotundifolia*, 2n = 40). This suggests that the divergence between the f and M haplotypes predates the divergence of the *Vitis* genus, consistent with the hypothesis of the single origin of dioecy in the *Vitis* genus.

As has been recently suggested (13), and supported by the results of this study, the evolution of dioecy in grapevines most likely evolved from a stepwise process of loss of hermaphroditism that began prior to species diversification in *Vitis*. It is clear that recombination is suppressed at the *Vitis* SDR, as no evidence of recombination was detected in the 129 wild genotypes, and our results show that LD in the SDR region is very high (Fig. 2). In some species, such as spinach, the SDR is located in a cold spot of recombination (22). This is not the case in *Vitis*, in which we find the SDR is located in a chromosomal region with a relatively normal recombination rate of 0.6 cm/Mb (23). Extremely high or low SNP density, as well as TE insertions, have been proposed as a consequence of the sex chromosome divergence, but may also contribute to recombination suppression (24–26). We compared the SNP density between the f and M haplotypes of the three male accessions *V. sylvestris* ‘DVIT3603.16', *V. arizonica* ‘b40-14', and *V. cinerea* ‘B9' and found that there is no significant difference in SNP density in these three genomes. However, the recombination breakpoints observed in the H1 and H2 hermaphroditic haplotypes occur in the region with moderate SNP density (*SI Appendix*, Fig. S12), perhaps explaining why the transaldolase region of the SDR has repeatedly been a site for recombination in the European clade.

The greatest structural variance between the M and f SDRs is the two TE clusters located at the 5′ end of *VvYABBY3* and 5′ end of *VviINP1*, the two sex-determining candidate genes. Numerous LTRs that are dated around 30 to 36 MYA further indicate that evolution of the SDR was accompanied by a pervasive TE movement (*SI Appendix*, Fig. S5). TE accumulation has also been observed to play an important role in determining the SDR in spinach and papaya (27, 28). In *Vitis*, the f haplotype is smaller and carries far fewer TE insertions compared with the M haplotype. Greater genetic divergence between species at the M allele suggests that the relaxed evolution of this locus in the wild is ongoing. These pieces of evidence suggest that the insertion of the TEs followed by the increased divergence plays a role in the recombination suppression in the wild *Vitis* SDR.

### Support for *VviINP1* as the Male-Sterile Candidate Gene.

When sex-linked SNPs were examined across the SDR, we found significant clustering of these SNPs associated with the female flower phenotype in the C region, the region where the candidate gene *VviINP1* resides. Markers spanning the 8 bp deletion in *VviINP1* discovered by Massonnet et al. (13) and those in linkage predicted the female phenotype at 100% accuracy in 167 accessions of grapevine, representing all *Euvitis* gene pools. All male accessions in this study carry one “functional” copy of *VviINP1* and one copy with the 8 bp deletion and predicted premature stop codon.

### Support for the Female Sterility Candidate Gene *VviYABBY3*.

Massonnet et al. (13) hypothesized that *VviYABBY3* is the most likely candidate gene for female sterility due to the association of M-linked SNPs and transcription factor binding sites whereas Badouin et al. (14) suggested *VviAPT3* is more likely. In our study, we tested these contrasting hypotheses by showing that M-linked SNPs associate with both the A and D regions of the SDR. However, our examination of the patterns of SNPs across hundreds of genotypes from all grapevine gene pools and the discovery of the H2 allele throw greater support to a single candidate, *VviYABBY3*. We demonstrated that all examples of hermaphroditic vines carry a critical recombination event in the sex-determining locus, combining an f-like SDR surrounding *VviYABBY3*, with an M-like SDR surrounding *VviINP1*. The most common form of H haplotype in this dataset, designated H1, was found in table, raisin, and wine grape germplasm. All H1 haplotype germplasms share a specific recombination in the 5′ end of a transaldolase gene in region B of the SDR. The H1 haplotype was observed in every possible combination with other alleles at the SDR: homozygous (H1/H1) and heterozygous (H1/f, H1/H2, and H1/M). The second, hermaphroditic haplotype, H2, is generated from what appears to be a double crossover recombination event with crossovers at the 3′ end of the transaldolase gene and also upstream of *VviAPT3*. The transaldolase recombination site is associated with the insertion of a TE cluster within the transaldolase gene itself, leading to an increased predicted gene length in H2 genotypes. The H2 haplotype, much like the H1 haplotype, has an f-like SDR surrounding *VviYABBY3* and an M-like SDR surrounding *VviINP1*. The sex-linked pattern of SNPs revealed by this double crossover recombination results in H2 genotypes in which the *VviAPT3* region can fluctuate and H2/f genotypes actually carry two f-like *VviAPT3*. As a result, comparing the recombination signatures of H1 and H2 haplotypes demonstrates that all hermaphroditic phenotypes carry an f-like *VviYABBY3* but can carry either f-like or M-like *VviAPT3*.

### Multiple Hermaphroditic Alleles Demonstrate Independent Evolutions of Hermaphroditism in Domesticated Grapevines.

While it has long been accepted that *V. vinifera* was domesticated from its wild ancestor *V. sylvestris*, the domestication history of grapevine is an ongoing topic of interest and national pride (18, 29). Using relatedness between *V. vinifera* and *V. sylvestris* accessions, Myles et al. (18) suggested a single origin for domesticated grapes with origins in the near East Caucasus region. However, western cultivars of *V. vinifera* also retain a signature suggesting introgression with wild *V. sylvestris* in this region. Distinct H haplogroups have been observed in a study of 73 wild and 39 hermaphrodite cultivated grapevines though results suggest a primary origin for H haplotypes in the Eastern Mediterranean region, with potential secondary centers elsewhere (10). In contrast to the single-origin theory, an analysis of chloroplast DNA polymorphisms in 1,201 *V. sylvestris* and cultivated grapevine suggested at least two origins for domesticated grapevine (19). In all cases, the H haplotype appears to have arisen from the M haplotype, similar to the results observed in our study. This result is perhaps intuitive given the critical recombination between M and f alleles to generate the unique patterns of H alleles observed in this study could only have occurred in male-flowering (M/f) genotypes.

Several specific pieces of evidence in this study support independent recombination events observed in the H1 and H2 gene pools. First, the breakpoint site of the recombination around the transaldolase gene is different in H1 and H2 haplotypes, and H2 haplotypes appear to be the result of a double crossover event. If the recombination between *VviYABBY3* and *VviINP1* giving rise to H1 and H2 alleles happened sequentially, then at least one side of the break should be highly similar to each other. Second, the divergence time in the *VviYABBY3* region and *VviINP1* region between the H1 and H2 alleles predate the divergence between *V. sylvestris* and *V. vinifera*. Third, we discovered an H2-specific TE inserted ∼6 MYA, which again suggests the divergence between H1 and H2 is much earlier than the presumed domestication of *V. vinifera* from *V. sylvestris*. This is consistent with a hypothesis of multiple (at least two) origins of grape hermaphroditism. We identified two unique patterns of recombination in only 363 genotypes. Even for interspecific hybrid accessions in this dataset (HYB1 and HYB2; Fig. 2), no additional hermaphroditic haplotypes were detected, leaving no evidence for independent origins of the H haplotype outside the European clade. However, it remains possible that other recombination events at the SDR exist among the estimated 6,000 to 12,000 named grape cultivars (30). The currently sequenced accessions of *V. sylvestris* come from the Caucasus region, in agreement with the hypothesis that this region played a key role in the domestication of grapevine (29). While we can associate the M haplotype of the sequenced *V. sylvestris* genomes with the H2 allele, the progenitor M haplotype for the widespread H1 allele can only be inferred from shotgun data. Greater efforts to sequence and analyze wild *V. sylvestris* is needed to help identify the potential origin of this haplotype.

### Numeric Genotypes Predict all Possible Flower Sex Haplotypes.

It typically takes at least two years for a grapevine to reach first bloom; thus, determining flower sex at the seedling stage would increase breeding efficiency by quickly identifying undesired flower sex phenotypes (typically, male or female vines). Previous efforts to develop molecular markers that predict flower sex in *Vitis* have resulted in two SSR markers with decent efficiency when used in domesticated grape backgrounds. These markers, VVIB23 and VVIAPRT3, are capable of predicting phenotypes for hermaphroditic vines when crossed with female vines (9, 20, 31). However, implementing these markers in crosses using wild grapevine germplasm has been less successful due to genetic divergence across the *Vitis* genus (32). With a greater understanding of the genetic architecture of the SDR revealed in this study, we can now see why these previous markers may fail. Specifically, these markers are on the two flanks of the SDR and do not distinguish the complexity of the A, B, C, and D regions as outlined in this study. Recently, primers were designed to evaluate the presence/absence of the 8 bp deletion in *VviINP1* (13), but these markers cannot distinguish between the H1 and H2 haplotypes. Breeders may decide that specific combinations of H alleles are preferable in their own programs. It is interesting to note that no H2/H2 genotypes were observed in this study and could suggest there are potential fitness consequences of different H combinations. The development of numeric genotypes gives breeders and researchers the tools needed to predict the flower sex genotype/phenotype for any of the 10 possible M, f, H1, and H2 genotype combinations.

## Conclusion

The evolution of hermaphroditism played a critical role in the domestication of grapevine. While there have been frequent investigations into the flower sex-determining locus in grapevine, until recently, the structure of the locus has remained elusive. However, the advancement of long-read whole-genome sequencing efforts in grapevine helped determine the origin of hermaphroditism in grapevine as the result of a rare recombination event. Using shotgun sequencing data from 363 hermaphroditic genotypes, we identified two unique patterns of recombination, giving rise to two distinct H haplotypes, H1 and H2. Structural differences between H1 and H2 haplotypes as well as divergence time estimates suggest diversification of the SDR occurred prior to domestication and support at least two independent evolutions of the hermaphroditic trait during the domestication history of grapevine.

## Materials and Methods

The details of the materials and methods are described in *SI Appendix*, *Supplementary Text*. To decipher the SDR in the *Vitis* genus, we examined the 12 whole-genome assemblies, including four genomes newly generated in this study, two bulk whole-genome sequencing with 13 female and 13 male *V. cinerea* accessions, genome-wide shotgun resequencing of 556 individuals (Dataset S2), transcriptome sequencing of 48 individuals representing diverse wild and cultivated germplasm resources (*SI Appendix*, Table S3), targeted Amplicon sequencing of 167 individuals (*SI Appendix*, Table S4), and rhAmpSeq sequencing of two biparental populations (*SI Appendix*, Figs. S10 and S11).

For wild dioecious species, we de novo assembled and scaffolded three wild genotypes: *V. cinerea* 'B9' (male), *V. rupestris* 'B38' (female), and *V. romanetii* 'C-166–043' (DVIT2732, female) (23). Regarding the H1/H2 *V. vinifera* cultivars, we sequenced the genomes of 'Chardonnay' and 'Riesling' using PacBio. Genome sequences for *V. riparia* 'Manitoba 37' (female) were provided from Patel et al. (16), and reference genome sequences for *V. arizonica* 'b40-14' (male), *V. sylvestris* 'DVIT3351.27' (male), *V. sylvestris *'DVIT3603.07' (female), *V. vinifera* cultivar 'Zinfandel', and the outgroup species *V. rotundifolia* 'Trayshed' (male) were obtained from Massonnet et al. (13) The genome of *V. vinifera* 'Carménère' was obtained from Minio et al. (33) All of the assemblies were annotated using the following pipeline. Structural annotation of the protein-coding genes in SDRs was based on the alignment of coding sequence (CDS) from 'Cabernet Sauvignon' SDRs and manually curated. RepeatMasker v.open-4.0.6 (34) was used with a custom *Vitis vinifera* ssp. *vinifera* repeat library (35) to identify repetitive and transposable elements.

For genome-wide shotgun resequencing data, we accessed data for 523 from the National Center for Biotechnology Information Sequence Read Archive (NCBI SRA) including 16 submitted in our previous study (23) and 17 from http://www.grapegenomics.com (36). The read depth of these samples ranges from 3× to 50×. The run accession, project number, sample name, and the species are included in Dataset S1. Reads were mapped and polymorphism were detected using 'Cabernet Sauvignon' f haplotype, denoted as CabSau_f, as the reference genome. Population structure was estimated using AIMs, which were determined considering F_st_ and population characterizing SNP (37).

Divergence time between H1 and H2 haplotypes was estimated based on conserved regions of the SDR with Mugsy (38). Conserved blocks within the C region of the SDR were concatenated due to the lack of historical recombination. The Akaike information criterion indicated that a Hasegawa–Kishono–Yano model +G+I was the best-fitted substitution model by jModelTest 2 v2.1.10 (39). The maximum likelihood (ML) phylogeny of these haplotypes was further calculated using RAxML v8.2.4 with a GTRCTA site rate substitution model (40). Genetic divergence for the ML phylogeny was estimated by Bayesian analysis with the software BEAST v.2.5.2 with a relaxed molecular clock for 80 × 10^6^ Markov chain Monte–Carlo cycles (41).

The Vitis International Variety Catalogue (https://www.vivc.de/) parent–offspring relationship data for the 556 accession with genome-wide shotgun sequences was used to clarify relationships between H1 and H2 haplotypes.

Flower sex phenotype; female, male, and hermaphroditic, was decomposed as the interaction of two factors, one determining male sterility/fertility, the other determining female sterility/fertility. Genome-wide association studies and allele-specific read depth association for flower sex were both conducted for male sterility and female sterility traits.

The candidate region with sex-linked SNPs was searched for primer using Primer3 (42) with target size 200bp to 270bp (Optimum 250bp) and *T*m between 57 and 64 °C. Marker details are listed in Dataset S4. We also designed a flower sex prediction pipeline which includes reads filtering, genotyping for sex-linked sites, and a phenotype prediction with Bayesian approach. The package of Vitis_flower_sex_predictor is publicly available at (https://bitbucket.org/cornell_bioinformatics/flower_sex_predictor) (43).

## Supplementary Material

Supplementary File

Supplementary File

Supplementary File

Supplementary File

Supplementary File

## Data Availability

Sequencing data have been deposited in National Center for Biotechnology Information Sequence Read Archive (NCBI SRA, PRJNA550461 and PRJNA281110) (44, 45). The genomes are public available at http://www.grapegenomics.com (36). The python package for flower sex prediction in the *Vitis* is available at bitbucket (https://bitbucket.org/cornell_bioinformatics/flower_sex_predictor/src/master/) (43).

## References

[1] C. Pratt, Reproductive anatomy in cultivated grapes-a review. Am. J. Enol. Vitic. 22, 92–109 (1971).

[2] P. This, T. Lacombe, M. R. Thomas, Historical origins and genetic diversity of wine grapes. Trends Genet. 22, 511–519 (2006). 16872714 10.1016/j.tig.2006.07.008

[3] A. Antcliff, Inheritance of sex in *Vitis*. Ann. Amelior. Plant. 30, 113–122 (1980).

[4] J. Battilana., Linkage mapping and molecular diversity at the flower sex locus in wild and cultivated grapevine reveal a prominent SSR haplotype in hermaphrodite plants. Mol. Biotechnol. 54, 1031–1037 (2013). 23532385 10.1007/s12033-013-9657-5 PMC3641292

[5] R. Ming, A. Bendahmane, S. S. Renner, Sex chromosomes in land plants. Annu. Rev. Plant Biol. 62, 485–514 (2011). 21526970 10.1146/annurev-arplant-042110-103914

[6] P. E. McGovern, Ancient Wine: The Search for the Origins of Viniculture (Princeton University Press, 2007).

[7] Y. Wan., A phylogenetic analysis of the grape genus (*Vitis* L.) reveals broad reticulation and concurrent diversification during neogene and quaternary climate change. BMC Evol. Biol. 13, 141 (2013). 23826735 10.1186/1471-2148-13-141 PMC3750556

[8] M. A. Dalbó., A gene controlling sex in grapevines placed on a molecular marker-based genetic map. Genome 43, 333–340 (2000). 10791822

[9] S. Riaz, A. F. Krivanek, K. Xu, M. A. Walker, Refined mapping of the Pierce’s disease resistance locus, PdR1, and sex on an extended genetic map of *Vitis rupestris* x *V. arizonica*. Theor. Appl. Genet. 113, 1317–1329 (2006). 16960717 10.1007/s00122-006-0385-0

[10] S. Picq., A small XY chromosomal region explains sex determination in wild dioecious *V. vinifera* and the reversal to hermaphroditism in domesticated grapevines. BMC Plant Biol. 14, 229 (2014). 25179565 10.1186/s12870-014-0229-z PMC4167142

[11] K. E. Hyma., Heterozygous mapping strategy (HetMappS) for high resolution genotyping-by-sequencing markers: A case study in grapevine. PLoS One 10, e0134880 (2015). 26244767 10.1371/journal.pone.0134880 PMC4526651

[12] Y. Zhou, M. Massonnet, J. S. Sanjak, D. Cantu, B. S. Gaut, Evolutionary genomics of grape (*Vitis vinifera* ssp. *vinifera*) domestication. Proc. Natl. Acad. Sci. U.S.A. 114, 11715–11720 (2017). 29042518 10.1073/pnas.1709257114 PMC5676911

[13] M. Massonnet., The genetic basis of sex determination in grapes. Nat. Commun. 11, 2902 (2020). 32518223 10.1038/s41467-020-16700-z PMC7283251

[14] H. Badouin., The wild grape genome sequence provides insights into the transition from dioecy to hermaphroditism during grape domestication. Genome Biol. 21, 223 (2020). 32892750 10.1186/s13059-020-02131-y PMC7487632

[15] A. Canaguier., A new version of the grapevine reference genome assembly (12X.v2) and of its annotation (VCost.v3). Genom. Data 14, 56–62 (2017). 28971018 10.1016/j.gdata.2017.09.002 PMC5612791

[16] S. Patel., Draft genome of the Native American cold hardy grapevine *Vitis riparia* Michx. ‘Manitoba 37’. Hortic. Res. 7, 92 (2020). 32528704 10.1038/s41438-020-0316-2 PMC7261805

[17] N. Girollet., De novo phased assembly of the *Vitis riparia* grape genome. Sci. Data 6, 1–8 (2019). 31324816 10.1038/s41597-019-0133-3 PMC6642119

[18] S. Myles., Genetic structure and domestication history of the grape. Proc. Natl. Acad. Sci. U.S.A. 108, 3530–3535 (2011). 21245334 10.1073/pnas.1009363108 PMC3048109

[19] R. Arroyo-García., Multiple origins of cultivated grapevine (*Vitis vinifera* L. ssp. *sativa*) based on chloroplast DNA polymorphisms. Mol. Ecol. 15, 3707–3714 (2006). 17032268 10.1111/j.1365-294X.2006.03049.x

[20] I. Fechter., Candidate genes within a 143 kb region of the flower sex locus in *Vitis*. Mol. Genet. Genomics 287, 247–259 (2012). 22258113 10.1007/s00438-012-0674-z

[21] Z.-Y. Ma., Phylogenomics, biogeography, and adaptive radiation of grapes. Mol. Phylogenet. Evol. 129, 258–267 (2018). 30195477 10.1016/j.ympev.2018.08.021

[22] S. Takahata., Comparison of spinach sex chromosomes with sugar beet autosomes reveals extensive synteny and low recombination at the male-determining locus. J. Hered. 107, 679–685 (2016). 27563071 10.1093/jhered/esw055

[23] C. Zou., Haplotyping the *Vitis* collinear core genome with rhAmpSeq improves marker transferability in a diverse genus. Nat. Commun. 11, 413 (2020). 31964885 10.1038/s41467-019-14280-1 PMC6972940

[24] D. Charlesworth, Evolution of recombination rates between sex chromosomes. Philos. Trans. R. Soc. Lond. B Biol. Sci. 372, 20160456 (2017). 29109220 10.1098/rstb.2016.0456 PMC5698619

[25] B. L. S. Furman., Sex chromosome evolution: So many exceptions to the rules. Genome Biol. Evol. 12, 750–763 (2020). 32315410 10.1093/gbe/evaa081 PMC7268786

[26] S. Ponnikas, H. Sigeman, J. K. Abbott, B. Hansson, Why do sex chromosomes stop recombining? Trends Genet. 34, 492–503 (2018). 29716744 10.1016/j.tig.2018.04.001

[27] J.-K. Na, J. Wang, R. Ming, Accumulation of interspersed and sex-specific repeats in the non-recombining region of papaya sex chromosomes. BMC Genomics 15, 335 (2014). 24885930 10.1186/1471-2164-15-335 PMC4035066

[28] S.-F. Li., The landscape of transposable elements and satellite DNAs in the genome of a dioecious plant spinach (*Spinacia oleracea* L.). Mob. DNA 10, 3 (2019). 30675191 10.1186/s13100-019-0147-6 PMC6337768

[29] A. Sivan., Genomic evidences support an independent history of grapevine domestication in the levant. bioRxiv [Preprint] (2020). 10.1101/2020.07.11.198358. (Accessed 12 July 2020).

[30] OIV, Distribution of the World’s grapevine varieties (International Organisation of Vine and Wine, 2017), p. 54. https://www.oiv.int/public/medias/5888/en-distribution-of-the-worlds-grapevine-varieties.pdf. Accessed 2 April 2021.

[31] J. L. Coito., *VviAPRT3* and *VviFSEX*: Two genes involved in sex specification able to distinguish different flower types in *Vitis*. Front. Plant Sci. 8, 98 (2017). 28197167 10.3389/fpls.2017.00098 PMC5281589

[32] S. Yang., A next-generation marker genotyping platform (AmpSeq) in heterozygous crops: A case study for marker-assisted selection in grapevine. Hortic. Res. 3, 16002 (2016). 27257505 10.1038/hortres.2016.2 PMC4879517

[33] A. Minio, M. Massonnet, R. Figueroa-Balderas, A. Castro, D. Cantu, Diploid genome assembly of the wine grape Carménère, G3 (Bethesda) 9, 1331–1337 (2019). 30923135 10.1534/g3.119.400030 PMC6505170

[34] A. F. A. Smit, R. Hubley, P. Green, RepeatMasker Open-4.0. 2013–2015 (2015). http://www.repeatmasker.org. Accessed 2 April 2021.

[35] A. Minio., Iso-seq allows genome-independent transcriptome profiling of grape berry development. G3 (Bethesda) 9, 755–767 (2019). 30642874 10.1534/g3.118.201008 PMC6404599

[36] D. Cantu, A web portal with genomic data and analysis tools for wild and cultivated grapevines. grapegenomics.com. http://www.grapegenomics.com/. Deposited 9 June 2020.

[37] R. Das, R. Roy, N. Venkatesh, Using ancestry informative markers (AIMs) to detect fine structures within Gorilla populations. Front. Genet. 10, 43 (2019). 30800141 10.3389/fgene.2019.00043 PMC6375890

[38] S. V. Angiuoli, S. L. Salzberg, Mugsy: Fast multiple alignment of closely related whole genomes. Bioinformatics 27, 334–342 (2011). 21148543 10.1093/bioinformatics/btq665 PMC3031037

[39] D. Darriba, G. L. Taboada, R. Doallo, D. Posada, jModelTest 2: More models, new heuristics and parallel computing. Nat. Methods 9, 772 (2012). 10.1038/nmeth.2109 PMC4594756 22847109

[40] A. Stamatakis, RAxML version 8: A tool for phylogenetic analysis and post-analysis of large phylogenies. Bioinformatics 30, 1312–1313 (2014). 24451623 10.1093/bioinformatics/btu033 PMC3998144

[41] A. J. Drummond, M. A. Suchard, D. Xie, A. Rambaut, Bayesian phylogenetics with BEAUti and the BEAST 1.7. Mol. Biol. Evol. 29, 1969–1973 (2012). 22367748 10.1093/molbev/mss075 PMC3408070

[42] T. Koressaar, M. Remm, Enhancements and modifications of primer design program Primer3. Bioinformatics 23, 1289–1291 (2007). 17379693 10.1093/bioinformatics/btm091

[43] C. Zou, Q. Sun, Vitis flower sex predictor. flower sex predictor. https://bitbucket.org/cornell_bioinformatics/flower_sex_predictor/src/master/. Deposited 16 November 2020.

[44] Y. Zhou, M. Massonnet, J. S. Sanjak, D. Cantu, B. S. Gaut. Data from "Evolutionary genomics of grape (Vitis vinifera ssp. vinifera) domestication." Sequence Read Archive. https://www.ncbi.nlm.nih.gov/bioproject/?term=PRJNA550461. Deposited 24 June 2019. 10.1073/pnas.1709257114 PMC5676911 29042518

[45] C. Zou., Data from "Haplotyping the Vitis collinear core genome with rhAmpSeq improves marker transferability in a diverse genus." Sequence Read Archive. https://www.ncbi.nlm.nih.gov/bioproject/?term=PRJNA281110. Deposited 1 January 2020. 10.1038/s41467-019-14280-1 PMC6972940 31964885

