# Supplementary material for: Multiple independent recombinations led to hermaphroditism in grapevine
Source: Proc Natl Acad Sci U S A. 2021 Apr 9;118(15):e2023548118. doi: 10.1073/pnas.2023548118 (PMC8053984; doi:10.1073/pnas.2023548118)
Supplement: Supplementary File [file pnas.2023548118.sapp.pdf]

PNAS
www.pnas.org

Supplementary Information for

Multiple independent recombinations led to hermaphroditism in grapevine

Cheng Zou[1]*, Mélanie Massonnet[2], Andrea Minio[2], Sagar Patel[3,4,5], Victor Llaca[6], Avinash Karn[7], Fred Gouker[7, a], Lance Cadle-Davidson[8], Bruce Reisch[7], Anne Fennell[3], Dario Cantu[2], Qi Sun[1]*, Jason P. Londo[8]*

* corresponding authors: Cheng Zou, Qi Sun and Jason P. Londo
Email: cz355@cornell.edu; qisun@cornell.edu; jason.londo@usda.gov

This PDF file includes:

Supplementary text
SI References
Figures S1 to S12
Tables S1 to S4
Legends for Datasets S1 to S4

Other supplementary materials for this manuscript include the following:

Datasets S1 to S4

Supplementary Information Text

Genome and transcriptome resources: DNA collection, genome assembly and annotation

In order to have a better understanding of the flower sex determination in the *Vitis* genus, we examined the grape SDR using the whole-genome assembly of 12 genotypes, including 4 genomes sequenced and assembled in this study, genome-wide shotgun resequencing of 556 individuals, targeted Amplicon sequencing of 167 individuals and transcriptome sequencing of 48 individuals which represents a large diversity of wild and cultivated germplasm resources.

For wild dioecious species, we *de novo* assembled and scaffolded three wild genotypes; *V. cinerea* 'B9' (male), *V. rupestris* 'B38' (female), and *V. romanetii* 'C-166-043' (DVIT2732, female) (1). Regarding the H1/H2 *V. vinifera* cultivars, we sequenced the genomes of Chardonnay and Riesling using PacBio reads. Genome sequences were provided for *V. riparia* 'Manitoba 37' (female) from Patel et al (2). Additionally, reference genome sequences for *V. arizonica* 'b40-14' (male), *V. sylvestris* DVIT3351.27(male), *V. sylvestris* DVIT3603.07 (female), *V. vinifera* cultivar 'Zinfandel', and the outgroup species *V. rotundifolia* 'Trayshed' (male) were obtained from Massonnet *et al*. (2020) (3). The genome of *V. vinifera* 'Carménère' was obtained from Minio *et al*. (2019) (4).

The draft genome of *V. cinerea* 'B9' was constructed using SMRT sequencing in our previous publication (1). In this study, we further improve the assembly using optical maps. Young, rapidly expanding leaf tissue was collected from *V. cinerea* 'B9' (PI588154, https://npgsweb.ars-grin.gov/gringlobal/accessiondetail.aspx?id=1003891), placed in a 50ml conical falcon tube, and immediately frozen in liquid nitrogen. Ultra-high-molecular-weight (uHMW) genomic DNA was extracted using the Bionano Plant DNA Isolation kit (https://bionanogenomics.com/support-page/plant-dna-isolation-kit/) following a modification of the Bionano Genomics high-polyphenolic protocol (1). The resulting agarose-embedded DNA was recovered by digesting the agarose with Agarase, removing excess starch and debris by high speed (16,000 xg) centrifugation and subjecting the supernatant to drop dialysis against TE buffer.

To assemble the chromosome- and chromosome-arm level optical maps, uHMW DNA was labeled and stained using the Bionano Genomics Direct Label and Stain (DLS) method as previously described (5). Unlike previous mapping technologies, Bionano DLS enables non-destructive, single fluorophore labeling of DNA molecules at CTTAAG sites. Labeled and stained DNA molecules were separated, stretched, imaged and digitized using a Bionano Saphyr system. One subset of 515,727 molecules (174.765 Gb total length) with a minimum length of 250 Kb and N50 334 Kb was used for assembly into maps using the Bionano Solve software as previously described (6). The

assembly was performed without pre-assembly using the non-haplotype parameters, no CMPR cut and without extend-split.

Young leaves were collected from *Vitis vinifera* ‘Chardonnay’ clone FPS 04 and ‘Riesling’ clone FPS 24. The high-molecular-weight genomic DNA was isolated and evaluated as previously described (3, 4). SMRTbell libraries were prepared as described in Minio *et al*. (7) and sequenced on a PacBio Sequel system I using V3 chemistry (DNA Technology Core Facility, University of California, Davis). SMRT reads from Riesling cultivar were assembled as described in Minio *et al*. (4) using a custom FALCON-Unzip pipeline (https://github.com/andreaminio/FalconUnzip-DClab).

A targeted assembly was conducted for the SDR of ‘Chardonnay’. PacBio reads from Zhou *et al*. (2019; BioProject ID PRJNA550461) were concatenated with the newly sequenced SMRT reads. Reads were mapped on ‘Riesling’ primary scaffolds using Minimap2 v.2.12-r847-dirty with the parameters: -ax map-pb --MD --cs -L (8). Reads mapping on the two ‘Riesling’ primary scaffolds containing the H1 and H2 SDRs, respectively, were selected and then assembled following the custom FALCON-Unzip pipeline mentioned above(4). As a control, the same approach was performed using the ‘Carménère’ PacBio reads (Bioproject PRJNA517468). This lead to the assembly of a single SDR which was 99.98% identical with the SDR from ‘Carménère’ genome assembly (4).

All of the assemblies were annotated using the following pipeline. Structural annotation of the protein-coding genes in SDRs was based on the alignment of CDS from ‘Cabernet Sauvignon’ SDRs and manually curated. RepeatMasker v.open-4.0.6 (9) was used with a custom *Vitis vinifera* ssp. *vinifera* repeat library (7) to identify repetitive and transposable elements. A schematic representation of the haplotypes was made using the Bioconductor package v.1.20.0 (10). For genome-wide shotgun resequencing data, we accessed data for 523 from the NCBI SRA including 16 submitted in our previous study (1) and 17 from www.grapegenomics.com. The read depth of these samples ranges from 3× to 50×. The SRR index, project number, sample name, and the species are included in SI Appendix, Dataset S1.

Bulk whole-genome sequencing in *V. cinerea*

Young leaf tissue was collected from 13 female and 13 male *V. cinerea* accessions currently held at the USDA Plant Genetic Resources Unit in Geneva, NY. The list of accessions is provided in SI Appendix, Table S1. DNA was isolated using Qiagen DNeasy Plant Maxi t extraction kit (Qiagen, Valencia CA, USA). DNA concentrations were normalized and pooled by flower sex type for library construction. Sequencing was performed using an Illumina NextSeq 500 to produce 1x150 bp reads and ~20 Gb of raw sequence for each pool. After initial QC, reads were mapped to the *V. cinerea* ‘B9’

reference genome using BWA MEM with default settings (11). The average read depth was 13× and 9× for female bulk samples and male bulk samples respectively.

Short-read archive sample collection and variant calling

Whole-genome sequence data was compiled from a total of 556 accessions as mentioned above. Due to the high conservation of the f haplotype across the *Vitis* genus (12), and to increase interoperability of the results, we used the 'Cabernet Sauvignon' f haplotype, denoted as CabSau_f, as the reference genome for determining background variance and substitution. Paired reads were mapped to this reference using BWA MEM with default settings (11). The variance was called using the Sentieon DNA Pipeline for variant detection(13). PCR duplicates were removed and realignment was performed around indels. HaplotypeCaller was used to detect the variance (14). In total, 15,784,179 genome-wide variances (SNP and small indel sites) with Minor Allele Frequency (MAF) larger than 0.01 and missing rate less than 0.10 were kept for downstream analysis.

Population structure and population characterization

Vitis species genomes are diverse and highly heterozygous (12, 15–17). The available genome resources are biased toward representing domesticated grape cultivars. To prevent bias in detecting population structure from this data, ancestry informative markers (AIM) were determined considering Fst and population characterizing SNP (18). These markers were targeted to the gene space, which has been shown to be more conserved than other regions. Samples were randomly chosen (n = 40 each) to include representation from wild North American grapevines (NA), wild Asian grapevines (EA), and European grapevines (EU), based on passport and species information provided at submission. Potential AIM sites were chosen after filtering the data for the top 5,000 sites with Fst values found between pairs in the four populations and from sites that are highly correlated with the first 50 principal components for each population. PCA was calculated with variant weights using PLINK v1.90 for each population (19). After removing redundant positions, a total of 36,691 SNPs was chosen as AIM sites. FastStructure was used to determine population structure with K ranging from 2 to 5 (20). Individuals were assigned to populations with a probability greater than 0.99, which occurred when K was set to 4.

The current genetic model of sex determination in *Vitis* describes genetic dominance between alleles of male (M) > hermaphrodite (H) > female (f) (21, 22). Male-flowering individuals are expected to carry one female haplotype and one male haplotype at the sex locus (M/f). Female individuals carry two female haplotypes (f/f). Using this framework, male-associated variants and female-associated variants are expected to have increased minor allele frequency and violate Hardy Weinberg equilibrium. The allele frequency spectrum for each population was calculated based on the genome-wide mean and

standard deviation using a 10 kb sliding window and the z-score was calculated for each window. Deviation from Hardy Weinberg equilibrium was tested using a χ^2 goodness-of-fit test in R (v.3.5.0). SNPs that significantly deviate from HWE ($p < 0.05$) were summed for each 1kb window. The z-score for each window was calculated based on the genome-wide mean and standard deviation and then the *P*-value was calculated using pnorm function in R (v.3.5.0). Linkage disequilibrium was estimated using all sites with MAF>0.05 and missing rate less than 0.5. Sites were randomly thinned to 30% and R^2 within 1Mb window were calculated using PLINK v.1.90 (19).

Estimating divergence at the SDR

Divergence time between H1 and H2 haplotypes was estimated based on conserved regions of the sex-determining locus using the program Mugsy (23). Samples included; four M haplotypes from *V. cinerea*, *V. arizonica*, and *V. sylvestris*; *one female haplotype from V. rupestris* 'B38'; H1 haplotypes from 'Chardonnay', 'Riesling', 'Carménère' and 'Zinfandel'; two H2 haplotypes from 'Chardonnay', and 'Riesling'; and an unclassified haplotype from *V. rotundifolia* as the outgroup. A total of 15 conserved blocks that were larger than 1,000 bp (SI Appendix, Fig. S6) were identified across the SDR. Conserved blocks within the C region of the SDR were concatenated due to the lack of historical recombination observed for this region. After removing assembly gaps and poorly aligned positions using Gblocks (24), multiple sequence alignment was conducted using PRANK (25). The Akaike information criterion (AIC) indicated that a Hasegawa–Kishono–Yano (HKY) *model* +G+I was the best-fitted substitution model by jModelTest 2 v2.1.10 (26). The maximum likelihood(ML) phylogeny of these haplotypes was further calculated using RAxML v8.2.4 with a GTRCTA site rate substitution model (27). Genetic divergence for the ML phylogeny was estimated by Bayesian analysis with the software BEAST v.2.5.2 with a relaxed molecular clock for 80×10^6 Markov chain Monte–Carlo cycles (28). The 95% credibility interval statistics for nodes and branches were summarized after a burn-in of 25% of the total generations that were sampled every 5,000 generations using TreeAnnotator v.1.8.3 implemented in BEAST. Divergence time was calculated by constraining the crown age of the *Vitis* genus with a normal prior distribution of 46.9 MYA (29).

LTRs were detected using the LTRharvest algorithm integrated with GenomeTools package (30). The target site sequences (TSD) of intact LTRs were extracted and aligned using MUSCLE (31). The genetic distance between the two TSDs was estimated using Kimura's two-parameter model (32). The insertion time of intact LTRs was calculated as $T = K/2\mu$, where K is the genetic distance between the two TSDs and μ is the mutation rate. A mutation rate of 2.5×10^{-9} mutations per nucleotide per year was assumed as has been shown in previous studies.

Pedigree relationships between accessions and tracking of hermaphroditic alleles

To clarify the historical relationship between grape cultivars with either the H1 or H2 hermaphroditic haplotypes, we accessed the Vitis International Variety Catalogue (http://www.vivc.de/) and queried parent-offspring relationship data for the 539 genebank accessions as well as 17 samples sequenced locally. A list of grandparent-parent-offspring relationships was summarized as a pedigree network of the sampled cultivars in this study using the igraph package in R (33) (Figure 4, SI Appendix, Dataset S3).

Genome-wide association studies and allele-specific read depth association for flower sex

There are three types of flower sex phenotype in the *Vitis* genus: female, male, and hermaphroditic. Flower sex was decomposed as the interaction of two factors, one determining male sterility/fertility, the other determining female-sterility/fertility. Hermaphroditic is a combination of male-fertility and female-fertility. We conducted a genome-wide association study (GWAS) using TASSEL 5 and alleles with MAF>0.05 and missing rate less than 0.5 (34). The association between the variance and the phenotype was detected using a mixed linear model (MLM) considering both population structure and the kinship matrix as covariance (34).

To measure the impact of gene expression in determining flower sex, flower buds were harvested from 29 accessions from 9 wild species (*V. amurensis, V. cinerea, V. labrusca, V. acerifolia*, *V. palmata*, *V. riparia*, *V. rupestris*, *V. vulpina*, and *V. sylvestris*) and 13 hermaphroditic *V. vinifera* (domesticated) accessions, and 6 bulked female and male samples from a bi-parental population (SI Appendix, Fig. S7, Table S3). RNA was extracted from the flower buds of each accession using a Sigma Spectrum RNA kit (Sigma) at stage H, which is around one week before anthesis. The concentration and purity of total RNA were tested with the Synergy HT Nanodrop system (Biotek, Germany). For each sample, 500ng of total RNA was used for library construction with Illumina Truseq kit. Over 20 million paired-end reads were generated for each sample with Hiseq2000. Clean reads were mapped to the CabSau_f reference genome using STAR (35), and then reads were split into exon segments and the mapping qualities were adjusted for variant detection. The downstream variant detection was identical to Senteion DNA Pipeline for Variant Detection, except that the soft clipped based were excluded and a lower minimum phred-scaled confidence was set as 20 (13). The number of uniquely mapped reads that supported each reference allele and alternative allele were summarized using pysam (https://github.com/pysam-developers/pysam). The association between the standardized number of reads supporting reference/alternative allele and the phenotypes were detected using a Kruskal-Wallis rank sum test by R (v.3.5.0).

Marker development for Amplicon sequencing-based genotyping

In order to make the primers work for diverse species in the *Vitis* genus, the primers were designed using a genus-wide approach similar to what we previously described[4]. We excluded all the variances with MAF >0.05 in the primer design to decrease possible mismatch between the primers and the template. The MAF is calculated based on variances called from 556 accessions of WGS in the previous section. The candidate region with sex-linked SNPs was searched for primer using Primer3 (36) with target size between 200bp-270bp (Optimum 250bp) and annealing temperatures (*T*m) between 57–64 °C (Optimum 60 °C). The rhAmpseq markers were designed using the rhAmpSeq Design Tool developed by Integrated DNA Technologies, Inc. (IDT, Coralville, IA, USA). rhAmpSeq amplification, indexing, and pooling were conducted as described previously(1). In the Ampseq platform, two runs of PCR are performed, 1) amplify a multiplex of target-specific primers with linkers, and 2) amplify the sample-specific barcoding was performed (37). The amplicon from Ampseq or rhAmpSeq were pooled and sequenced with an Illumina MiSeq (Illumina, San Diego, CA, USA) with paired-end 2 x 150 bp mode. Reads were demultiplexed and genotyped using an in-house optimized pipeline (https://bitbucket.org/cornell_bioinformatics/amplicon).

Phenotype and Genotype prediction for the SDR

Because sex-linked sites violate Mendel's law of segregation, we first inferred the log-likelihood of genotype for each bi-allelic site for each sample independently without considering population genotype, which is an approach proposed by Li (38) and adopted in the estimation of PL (the phred-scaled genotype likelihoods) in the GATK HaplotypeCaller (14). For each sample, assuming the number of reads that covering one allele follows the binomial distribution with parameters *n* and *p*, abbreviated *B(n,p),* where n is the total number of reads that are uniquely aligned to this site, *p* is the probability that the reads supporting the alternative allele. Then we assume homologous reference genotype (denoted as 0) follows *B(n,0.001)*, the heterozygous genotype (denoted as 1) follows *B(n,0.5)*, and the homologous alternative genotype (denoted as 2) follows *B*(*n,0.999*). The log-likelihood of each genotype (0, 1, 2) given the read counts that we have observed is calculated by $-\log_{10}$ P(Data|genotype), then the genotype with highest likelihood is chosen for each site in the downstream analysis. To determine the genotype for the A, B, C, D region, we fit all the genotypes in one region into a linear regression with slope equals 0 and the intersect equals 0,1,2 respectively. The model with the smallest Least Squares Fitting were picked for each region. To determine the flower sex phenotype, we only need consider the genotype in A region and C region, when C region is homozygous reference genotype (f/f), the phenotype is male-sterile, when A region is not homozygous reference genotype (f/f), the phenotype is female-sterile. We used Bayes factor hypothesis testing comparing the null hypothesis that all sites are homozygous reference and the alternative hypothesis that all sites are heterozygous,

$$BF = \frac{p(data|H_1)}{p(data|H_0)}$$

where, H_0~$B(n_0,p_0)$, H_1~$B(n_1,p_1)$. $B(n,p)$ is another binomial distribution, in which the n denotes the total number of allelic sites considering all sites in this region, which is 2 fold of the total sites considered. And the *p* is the probability of the alternative allelic sites in this region. BF > 3 indicates substantial evidence supporting that the region examined is heterozygous, while BF<0.33 indicates substantial evidence supporting that the region is homozygous reference genotype. When using only one site to predict the flower sex, the BF ranges from 0.5 to 3 due to the small sample size. BF ranges from 0.5-1 to 1-3 only indicate anecdotal evidence supporting H0 or H1 in general, however in our prediction they are 100% consistent with the phenotype. Therefore, smaller BFs are still trustable when only using a few sites in the prediction. The package of Vitis_flower_sex_predictor is publicly available at (https://bitbucket.org/cornell_bioinformatics/flower_sex_predictor)

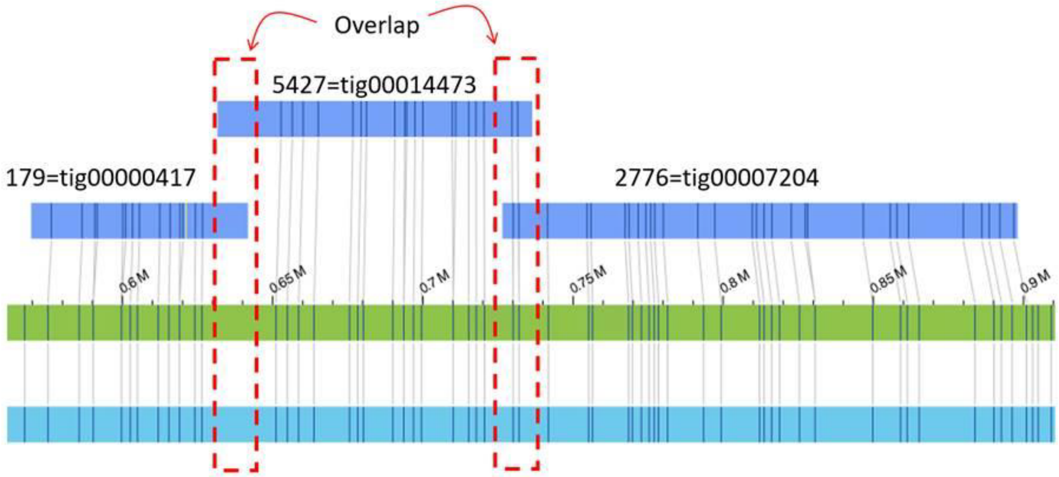

	Original BioNano Genome Map statistics	Original NGS sequences statistics	Hybrid scaffold FASTA statistics
Count	176	6576	143
Min length (Mbp)	0.214	0.005	0.094
Median length (Mbp)	2.278	0.048	2.429
Mean length (Mbp)	4.914	0.107	5.292
N50 length (Mbp)	11.857	0.226	13.260
Max length (Mbp)	29.963	2.616	29.659
Total length	864.902	702.289	756.822

Fig. S1. Hybrid scaffolding of *V. cinerea* 'B9' with BioNano Optical Mapping. Upper panel, the scaffolding of contig 2 with three contigs from the previous assembly. Lower panel, summary statistics of the scaffolding.

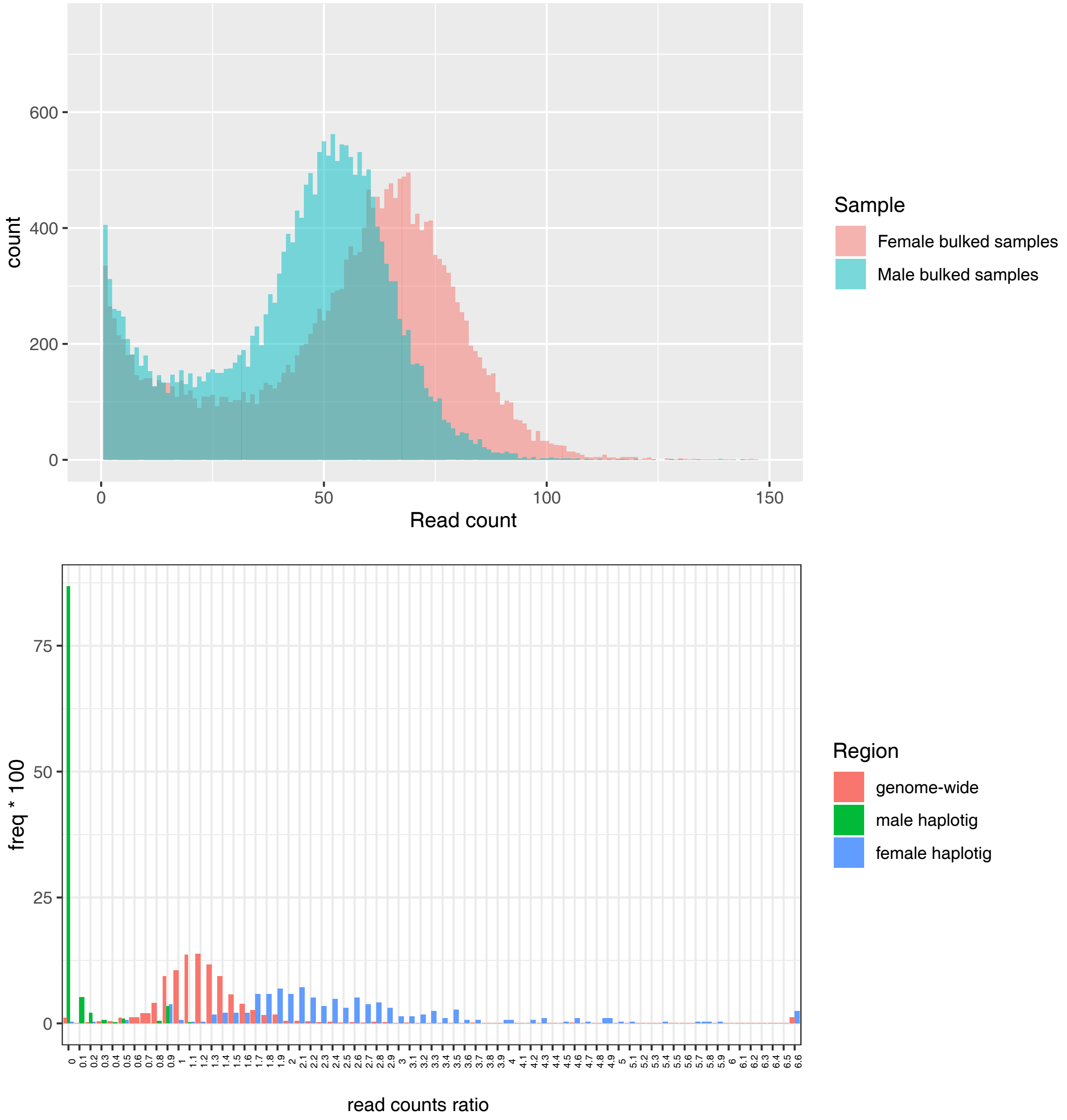

Fig. S2. Read counts in the bulked sample analysis. a. genome-wide read counts distribution in 500 bp windows. **b.** Distribution of the ratio of read counts in female bulked sample to male bulked sample on genome-wide region, female haplotype and male haplotype. These three distribution is significantly different between each other(two-sided Kolmogorov-Smirnov test, p<1e-12)

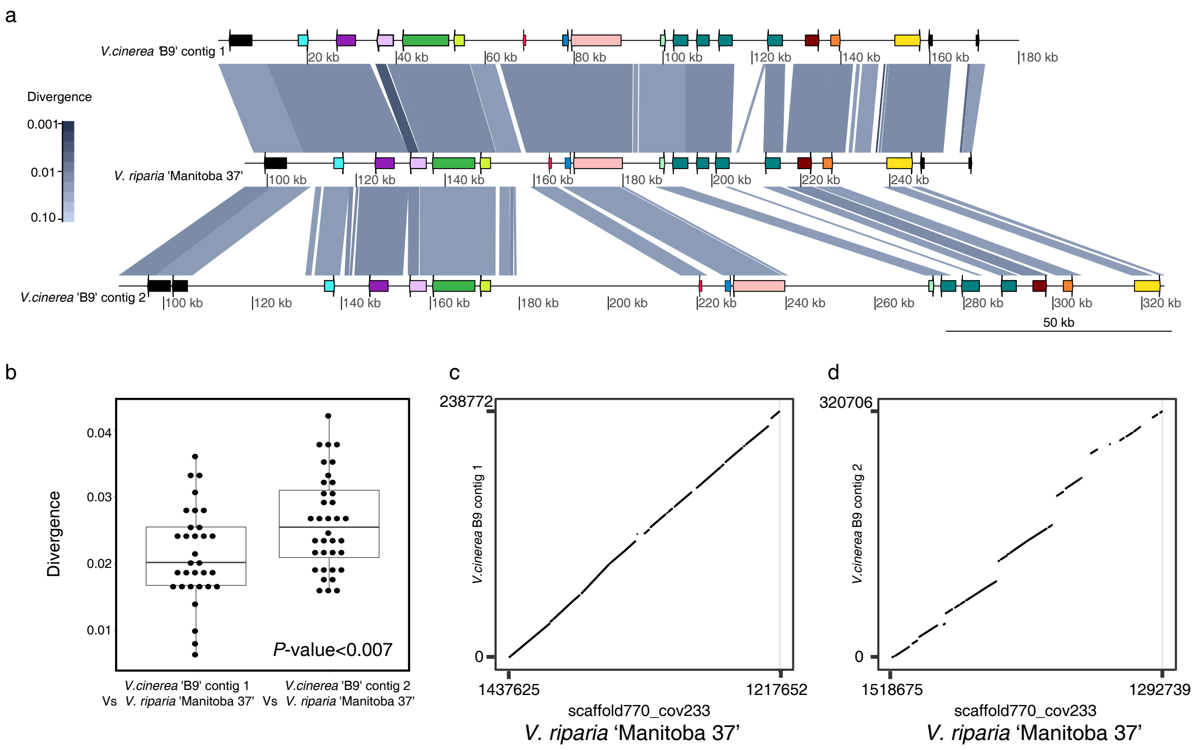

Fig. S3. Genome comparison of SDR between *V.cinerea* 'B9' contigs and *V. riparia* 'Manitoba 37'. a. genome alignment between SDR contigs in *V.cinerea* B9 and SDR in *V. riparia* 'Manitoba 37'. Genes are colored-coded as shown in figure 1, and grey blocks indicate the genetic similarity of the alignment. **b.** Genetic divergence between two SDR contigs in *V.cinerea* B9 and SDR in *V. riparia* 'Manitoba 37' are significantly different (unpaired t-test). **c.** Dot plot comparison indicating structure variance between two potential SDR contigs in *V.cinerea* 'B9' and SDR in *V. riparia* 'Manitoba 37'.

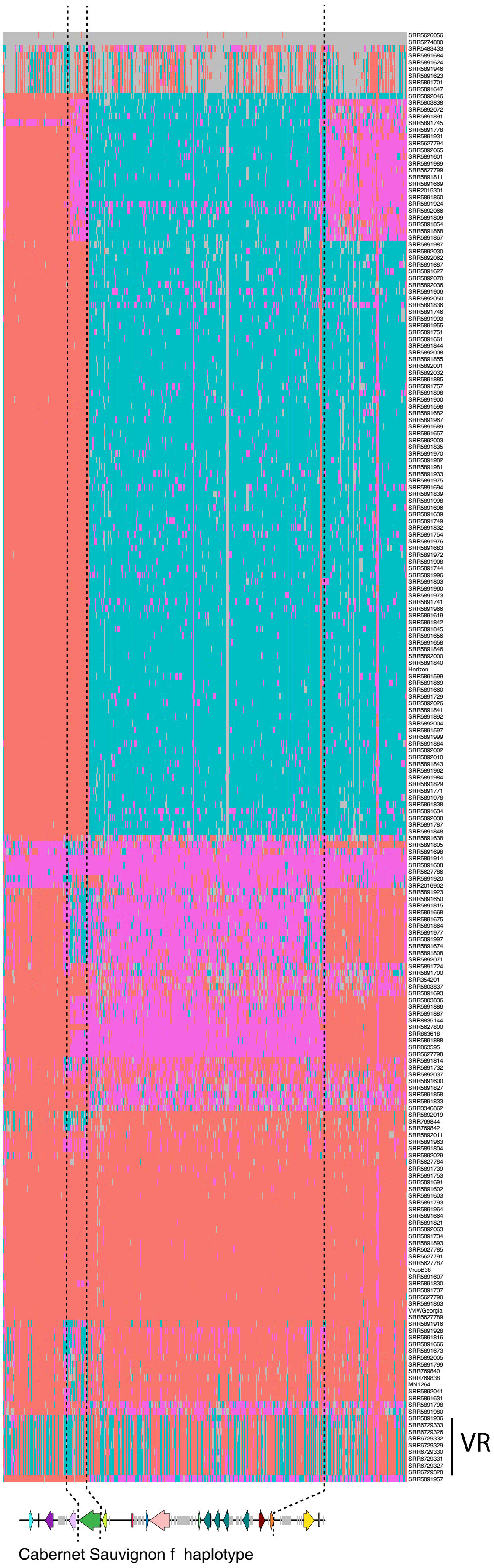

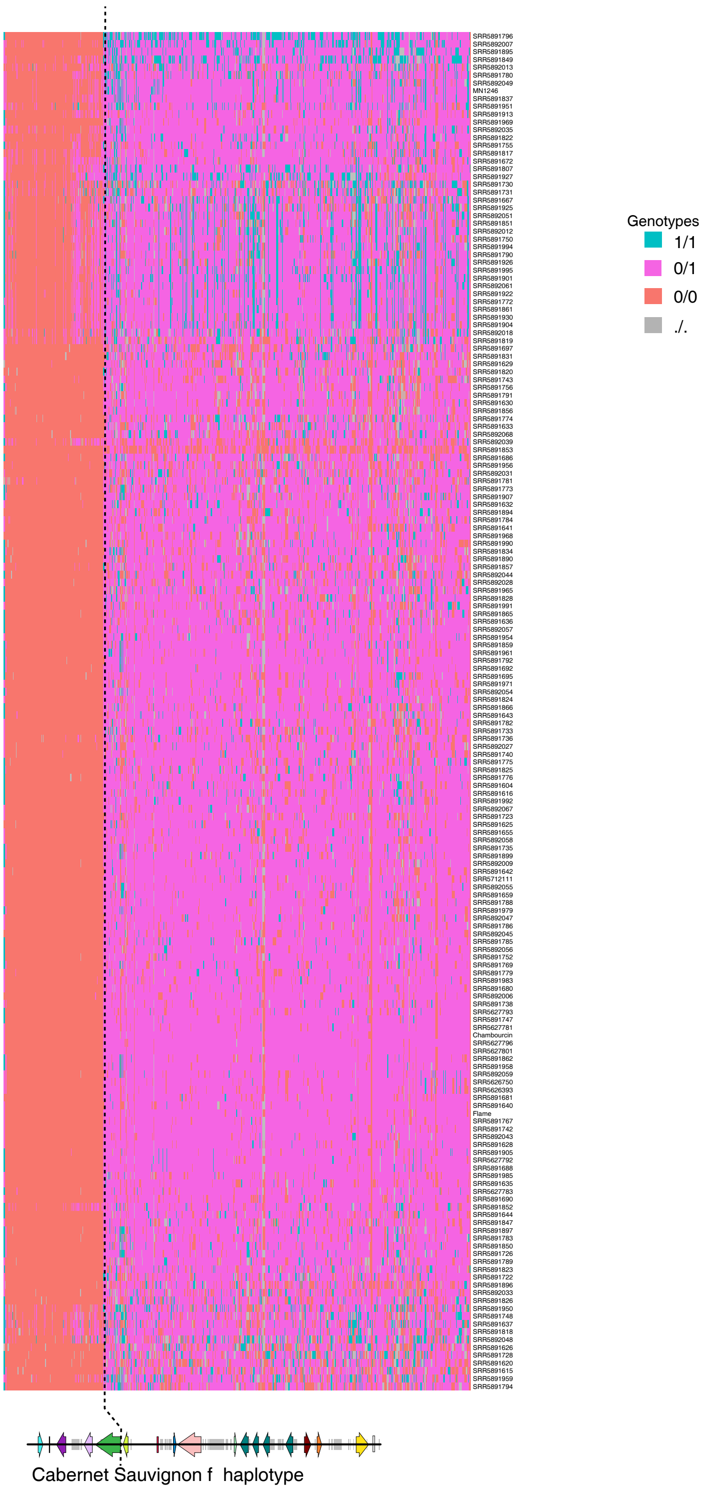

Fig. S4. Allelic state of the 1,066 sex-linked SNPs in 363 accessions with domesti-cated *V. vinifera* or mixed germplasm. Colors represent allelic state: red= homozygous reference allele (f allele), magenta = heterozygous, blue = homozygous alternative allele (M allele). Each row illustrates the genotype of one sample. Dashed black lines indicate detected recombination sites. VR indicates accessions from *Vitis rotundifolia*.

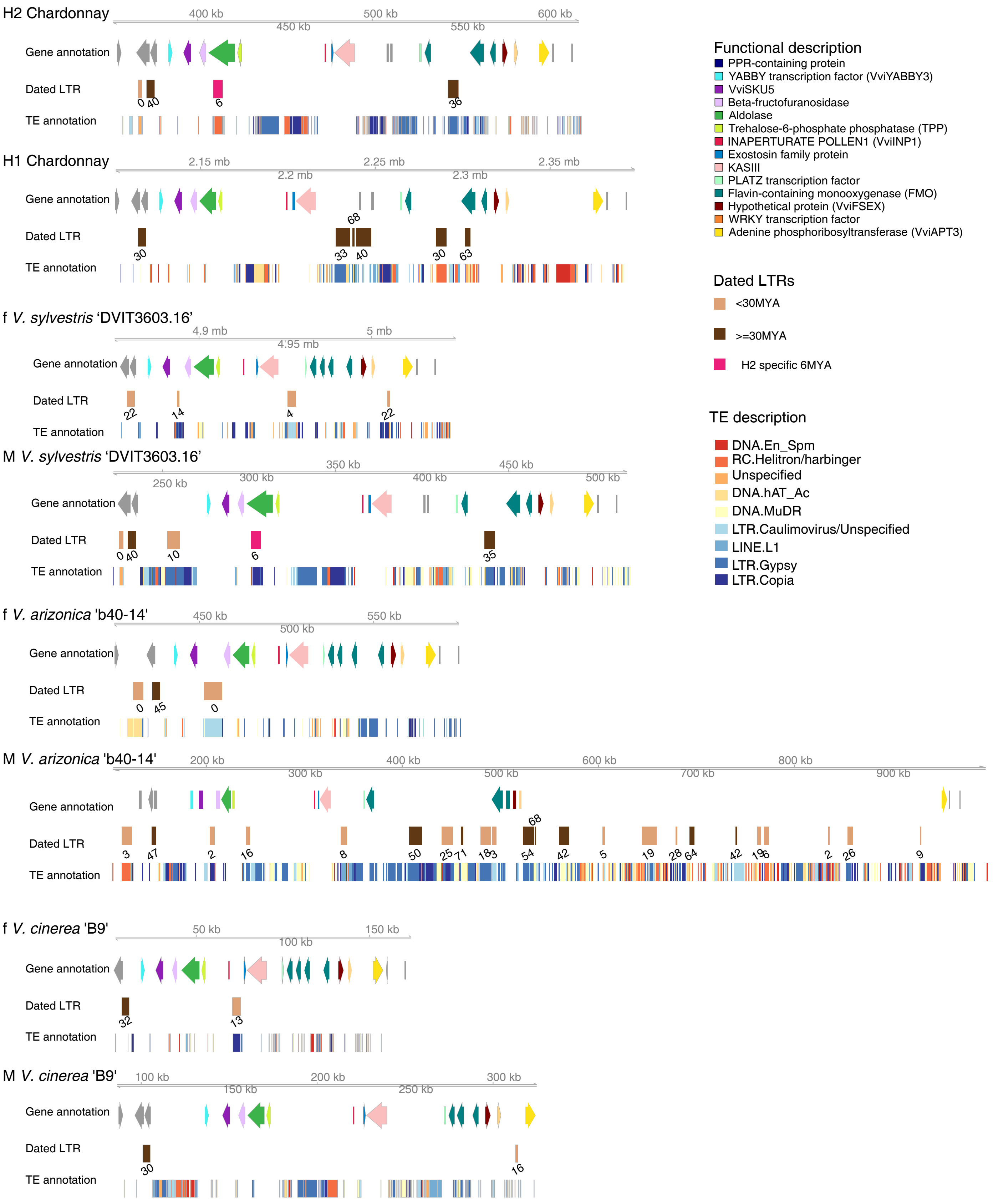

Fig. S5. TE annotation and LTRs dating in the SDR. For each observed haplotype, the genes are color-coded as in figure1, the LTRs were dated using LTRharvest and with the mutation rate of 2.5×10^{-9} mutations per nucleotide per year, the TEs were annotated using RepeatMasker with a grape curated database described in Minio et al. (2019).

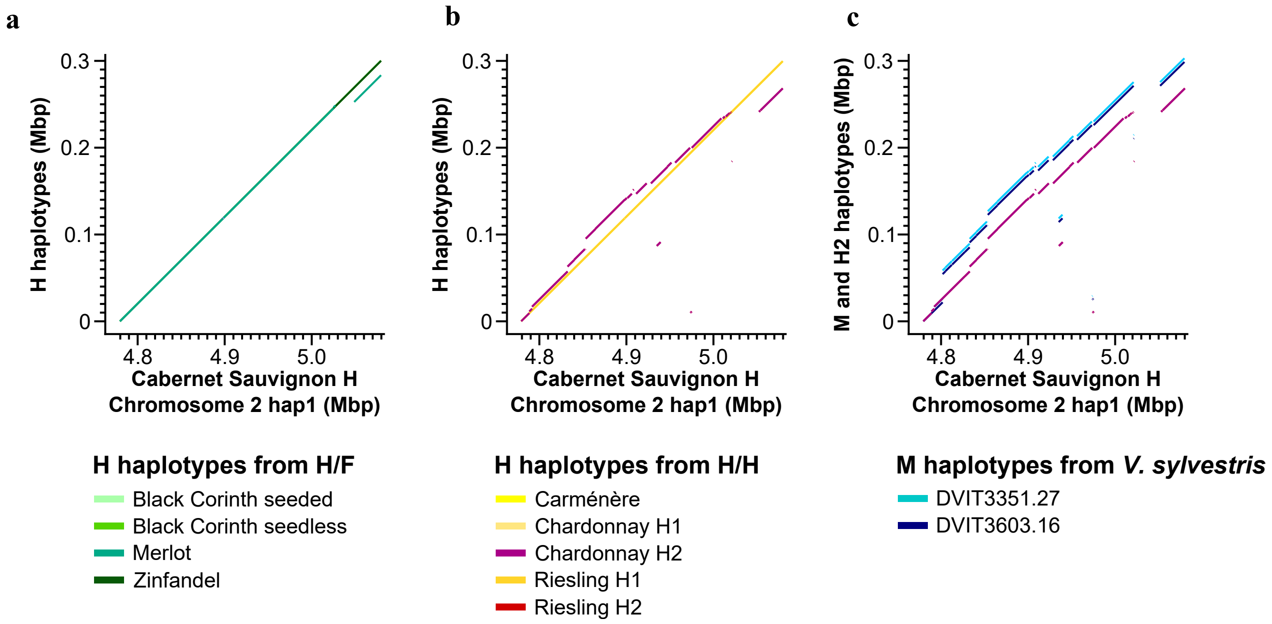

Fig. S6. H1 and H2 haplotype have different genome structures. a. Whole-sequence alignments of the H1 haplotype in H1/f grapevine and Cabernet Sauvignon H Chromosome 2 hap1 **b.** Whole-sequence alignments of the H2 haplotype in H1/H2 grapevine and Cabernet Sauvignon H Chromosome 2 hap1. **c.** M haplotypes in *V. sylvestris* 'DVIT3351.27' and *V. sylvestris* 'DVIT3603.16' are structurally similar to H2 haplotype.

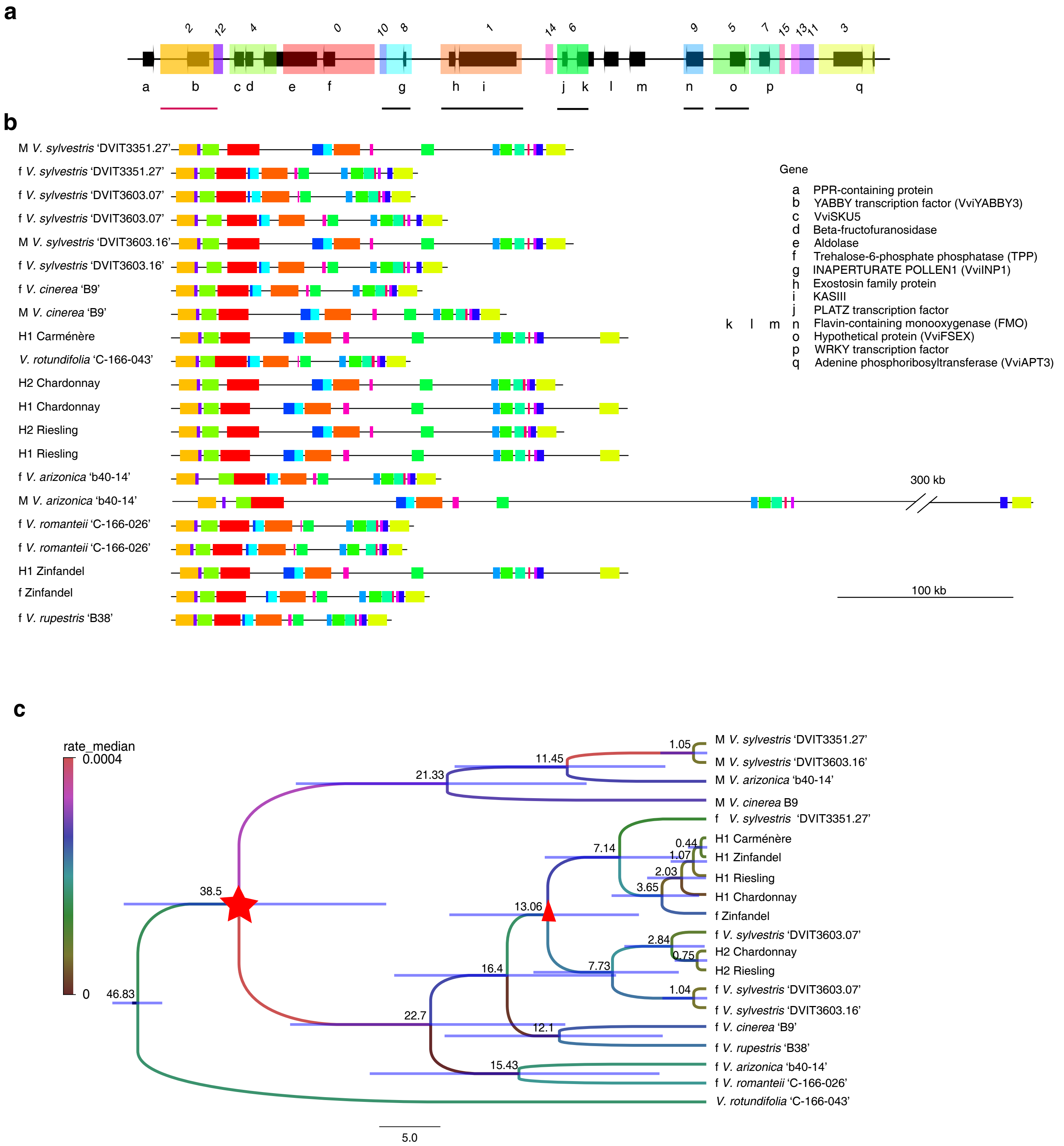

Fig. S7. Divergence time estimation based on conserved blocks in the SDR. a. Genome position of the conserved blocks and their comparative position of annotated genes using *V. vinifera* Cabernet Sauvignon chromosome 2 hap2 (f) as reference for whole-genome datasets. **b.** The coordinates of the conserved blocks in each genome **c.** The divergence time of M, f, H1, and H2 haplotypes estimated based on the conserved block around *VviYABBY3*. The red star indicates the presumed divergence of female and male (and H) haplotypes. The red triangle denotes the divergence of H1 and H2 haplotypes.

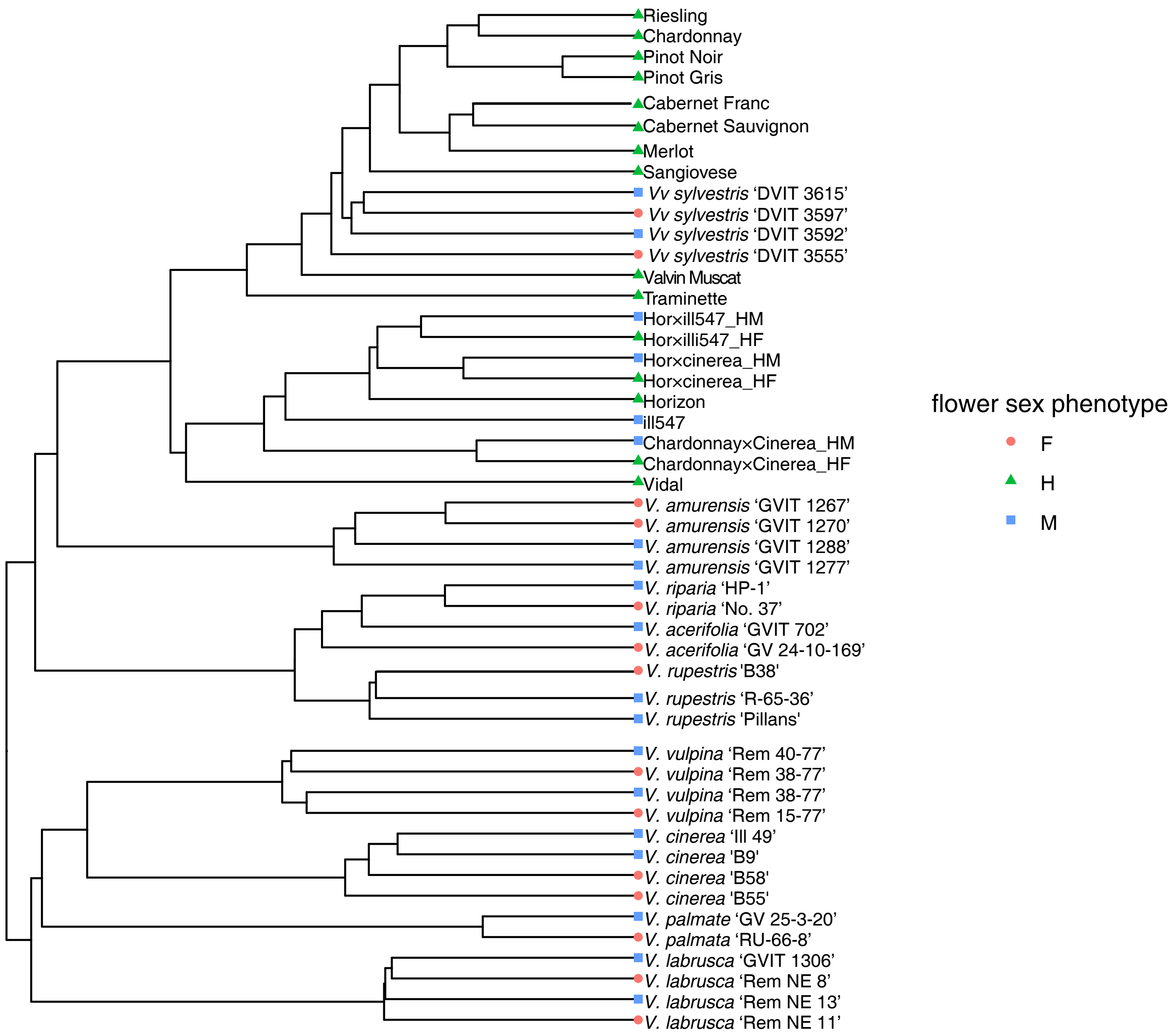

Fig. S8. Phylogeny of all wild and cultivated grapevines used in the RNA-seq analysis. The phylogeny is estimated using genome-wide SNP polymorphism using *V. vinifera* Cabernet Sauvignon chromosome 2 hap2 (f) reference with a hierarchical clustering algorithm of the IBS distance matrix in the SNPRelate package.

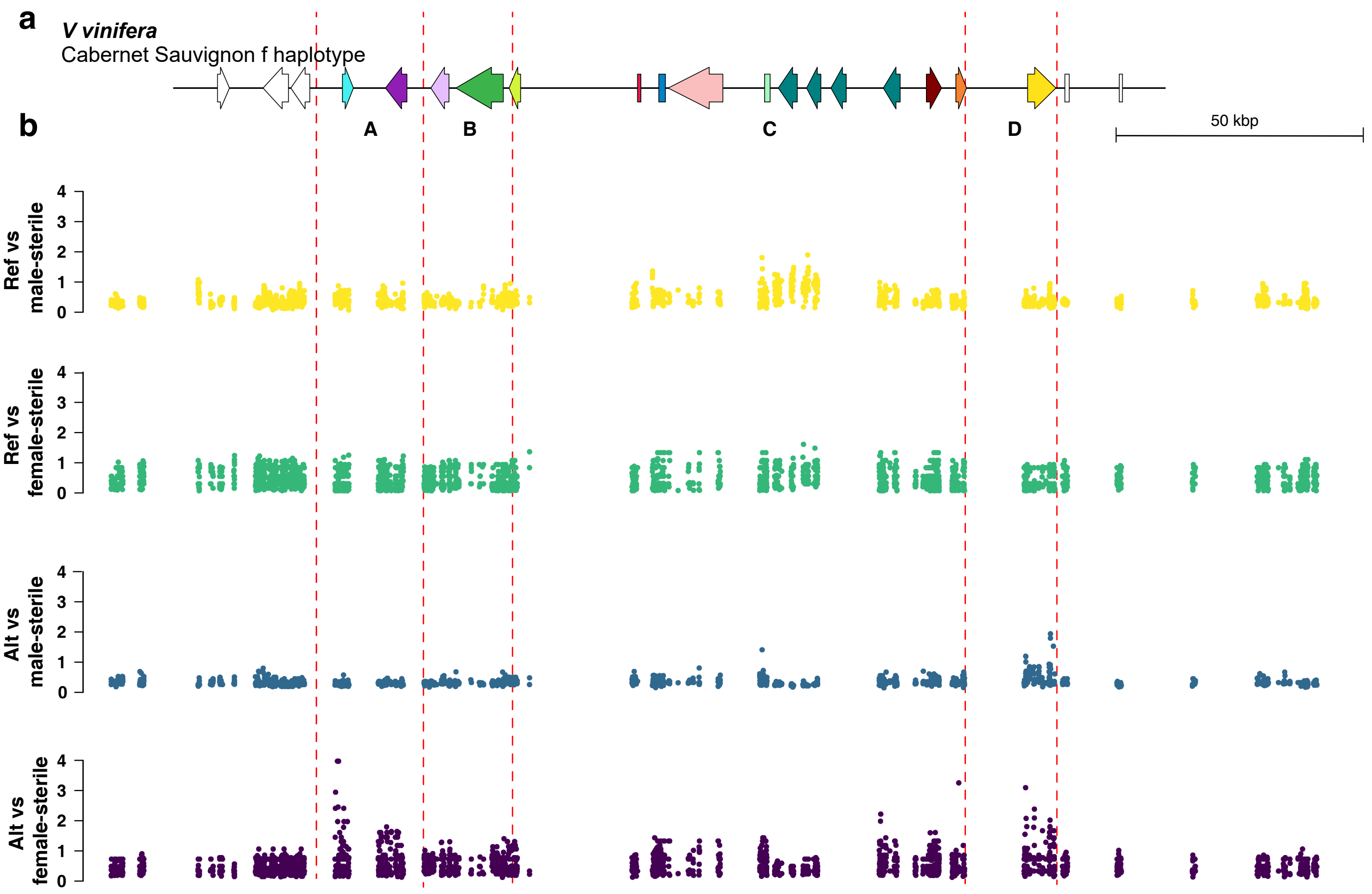

Fig. S9. The association between flower sex phenotype and allele-specific expression. a. Gene models for predicted genes located within the sex locus boundary. **b.** Dot plot demonstrating the association between flower sex phenotype and allele-specific expression Y-axis denotes the $\log_{10}$ *P*-value of the association.

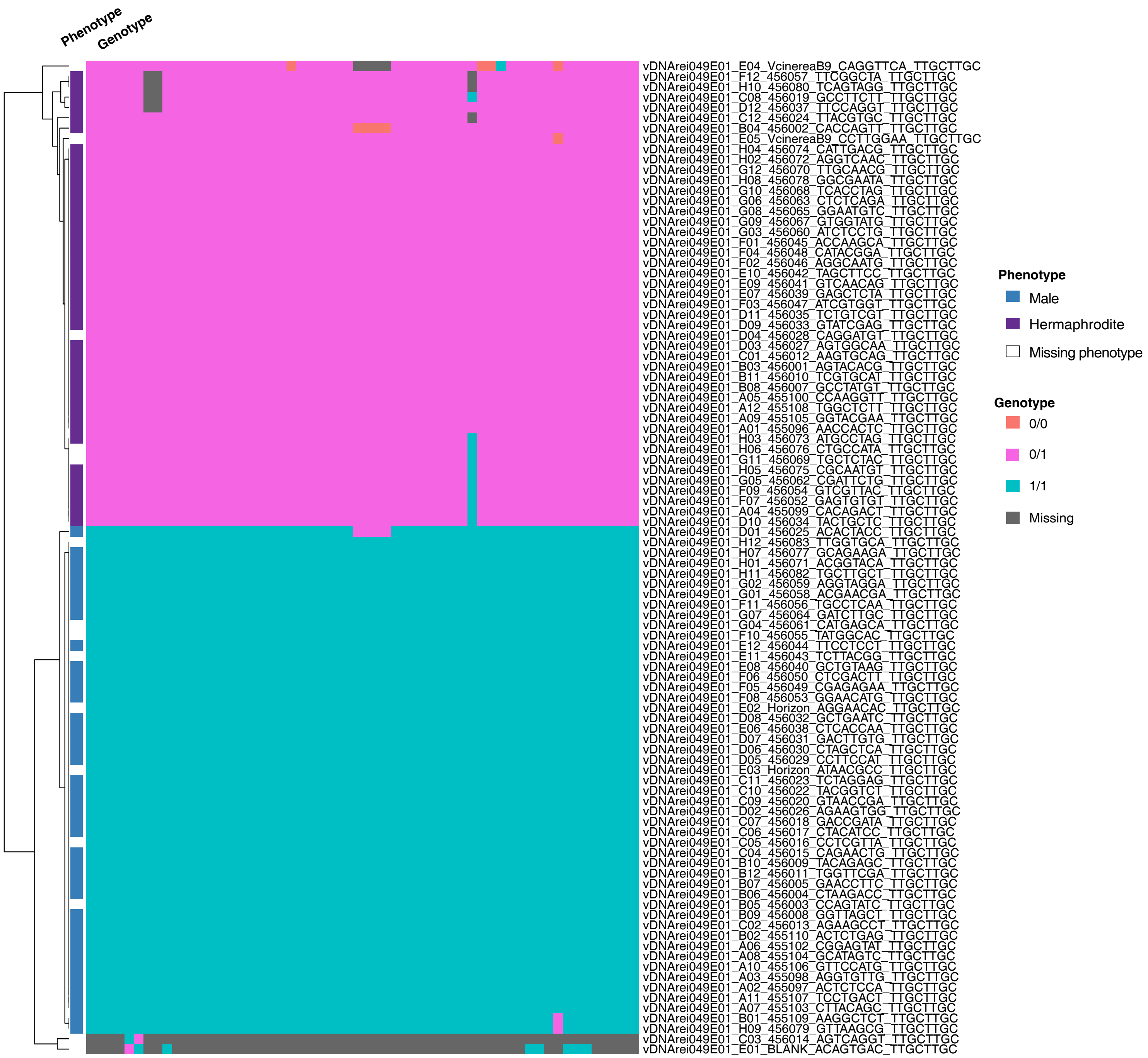

Fig. S10. Genotyping in C region using Ampseq markers and the flower sex phenotype of a F1 family Horizon (H/H) × *V. Cinerea* 'B9' (M/F). Let 0 denote the female allele, while 1 denotes the male/hermaphroditic allele. The offsprings with H/F are representing 0/1 genotype and have hermaphroditic flowers. The offsprings with M/H are representing 1/1 genotype and have male flowers.

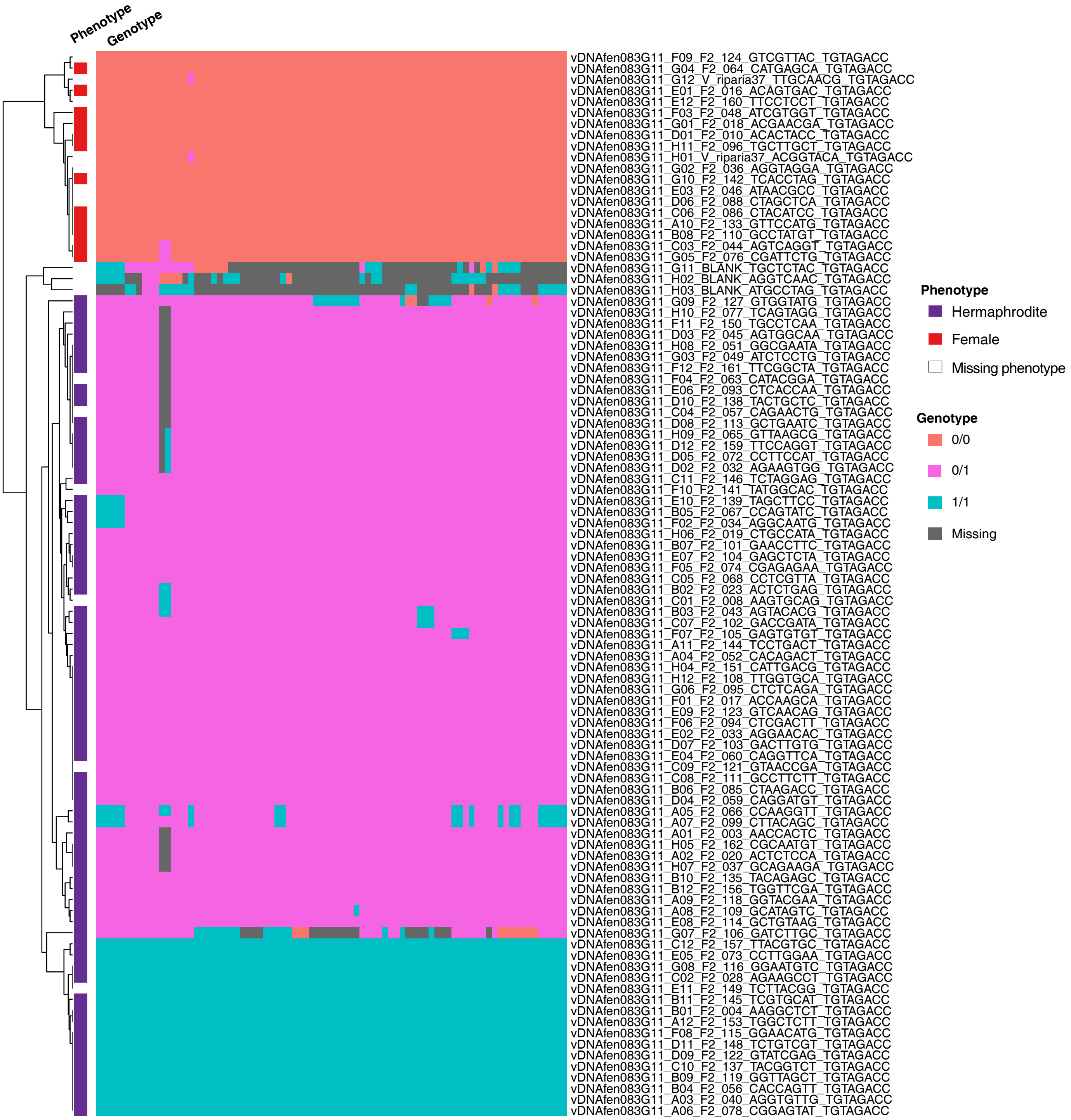

Fig. S11. Genotyping in C region using Ampseq markers and the flower sex phenotype of an F2 family 16_9_2 (H/f) × 16_9_2 (H/f). 16_9_2 is a grapevine from a cross between *V. riparia* and hybrid cultivar Seyval. Let 0 denote the female allele, while 1 denotes the male/hermaphroditic allele. The offsprings with H/H or H/f are representing 1/1 and 0/1 genotype respectively and have hermaphroditic flowers. The offspring with f/f are representing 0/0 genotype and having female flowers.

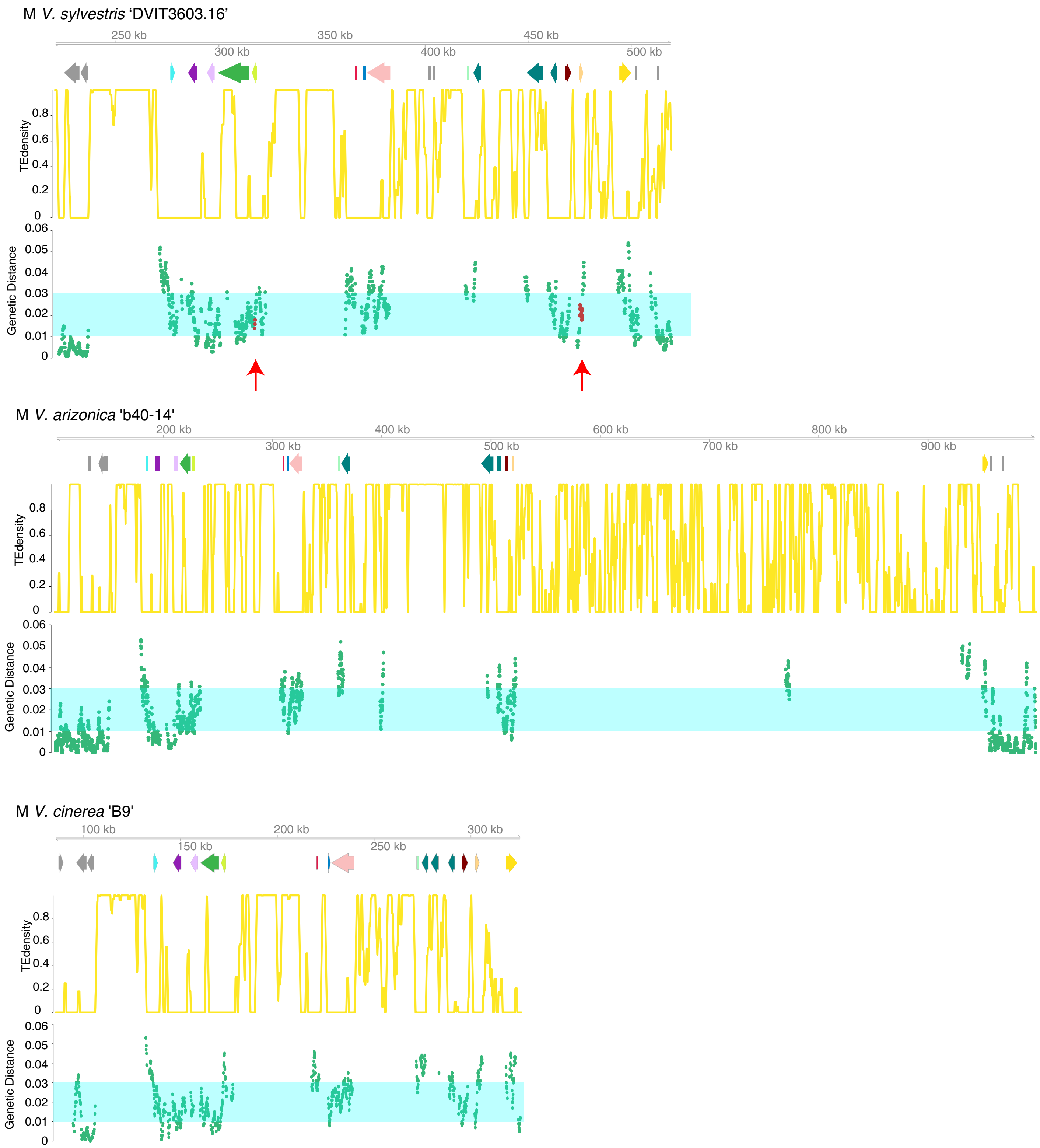

Fig. S12. TE density and the genetic distance between the M and f haplotype in three male grapevines. Annotated genes on the f haplotype were plot in the first track, the TE density and genetic distance was calculated for each 1000 bp window with 200bp step. The red arrows and dots indicate the position where the two recombinations in H2 happens.

SUPPLEMENTARY TABLES:

Table S1 Accessions collected in the bulked sample sequencing in *V. cinerea*

USDA ID	Accession Name	Species	Subspecies	Flower sex scale*
**PI_588199**	C-66-14	*V. cinerea*		1
**PI_588198**	Ill 46	*V. cinerea*		1
**PI_588352**	C-66-6	*V. cinerea*		1
**PI_588134**	Ill 45	*V. cinerea*		1
**PI_588219**	B 49	*V. cinerea*		1
**PI_588356**	GV 25-21-91	*V. cinerea*		1
**PI_588154**	B 9	*V. cinerea*		1
**PI_588197**	Ill 49	*V. cinerea*		1
**PI_588466**	Mazade	*V. cinerea*	*var. helleri*	1
**PI_588328**	Ill 66	*V. cinerea*		1
**PI_588372**	Ill 194-1	*V. cinerea*		1
**PI_588329**	Ill 24	*V. cinerea*		1
**PI_588625**	B 65-20	*V. cinerea*	*var. helleri*	1
**PI_588205**	GV 21-46-75	*V. cinerea*	*var. helleri*	5
**PI_588200**	Ill 55	*V. cinerea*		5
**PI_588222**	C-66-7	*V. cinerea*		5
**PI_588220**	B 55	*V. cinerea*		5
**PI_588217**	B 27	*V. cinerea*		5
**PI_588218**	B 47	*V. cinerea*		5
**PI_588186**	Ill 58	*V. cinerea*		5
**PI_588398**	Barrett 1	*V. cinerea*		5
**PI_588143**	Ill 41	*V. cinerea*		5
**PI_588575**	C-65-21	*V. cinerea*		5
**PI_588678**	JEG 2 SPR CR	*V. cinerea*		5
**PI_597232**	Ill 23	*V. cinerea*		5
**PI_588685**	JEG 3 IGLOO	*V. cinerea*		5

*Flower sex scale:1=Male; 2=Male to Hermaphrodite; 3=Hermaphrodite; 4=Female, with upright stamina; 5=Female.

Table S2. Summary statistics of genome assemblies of Carménère and Riesling

		V. vinifera **Carménère FPS02**		***V. vinifera*** **Riesling FPS24**	
**Sequencing**	Sequencing coverage (PacBio)	56 Gbp (112X)		59 Gbp (118X)	
	Best assembly read length cutoff (length_cutoff_pr) (bp)	7.5 kbp		21.5 kbp	
**Genome assembly**	**Assembly**	**Primary assembly**	**Haplotigs**	**Primary assembly**	**Haplotigs**
	Assembly length (bp)	622,795,289	420,345,460	741,809,687	323,202,626
	Number of sequences	1,411	7,969	528	3,244
	Average length (bp)	441,386	52,748	1,404,943	99,631
	Maximum length (bp)	5,905,621	743,383	12,495,899	2,705,535
	N50 length (bp)	1,039,379	89,565	2,970,313	205,547
	Total gap length (bp)	857,125	0	480,021	0
	Repetitive content (%)	49.60%	45.70%	49.7%	49.7%
**BUSCO**	Complete Single-Copy BUSCOs (%)	96.5%		98.4%	
	Fragmented BUSCOs (%)	0.9%		0.5%	
	Missing BUSCOs (%)	2.6%		1.1%	

Table S3. Individuals and bulked samples collected in RNA-seq analysis and flower sex phenotype prediction.

Sample_name	Species	USDA_ID	Haplotype	Phenotype	BF_A	BF_C
**Horizon**	hybrid	PI_588069	HH	H	9.09E-02	7.70E+51
**Ill547-1**	hybrid	PI_588327	MF	M	1.25E+01	2.71E+35
***V. acerifolia* GV 24-10-169**	*V. acerifolia*	PI_588324	FF	F	9.09E-02	5.53E-61
***V. acerifolia* GVIT 702**	*V. acerifolia*	PI_588393	MF	M	4.76E+00	2.07E+50
***V. amurensis* GVIT 1270**	*V. amurensis*	PI_588633	FF	F	5.00E-01	5.45E-49
***V. amurensis* GVIT 1277**	*V. amurensis*	PI_588635	MF	M	9.09E-02	1.74E-56*
***V. amurensis* GVIT 1267**	*V. amurensis*	PI_588631	FF	F	5.00E-01	5.47E-52
***V. amurensis* GVIT 1288**	*V. amurensis*	PI_588641	MF	M	1.33E+00	8.45E+43
***V. cinerea* Ill 49**	*V. cinerea*	PI_588197	MF	M	1.25E+01	8.13E+17
***V. cinerea* B 55**	*V. cinerea*	PI_588220	FF	F	9.09E-02	4.32E-58
***V. cinerea* B 58**	*V. cinerea*	PI_588221	FF	F	9.09E-02	4.11E-53
***V. cinerea* B 9**	*V. cinerea*	PI_588154	MF	M	1.33E+00	2.19E+29
***V. labrusca* Rem NE 8**	*V. labrusca*	PI_483150	FF	F	5.00E-01	1.47E-38
***V. labrusca* Rem NE 11**	*V. labrusca*	PI_483152	FF	F	9.09E-02	3.11E-42
***V. labrusca* Rem NE 13**	*V. labrusca*	PI_483154	MF	M	3.20E+01	4.76E+36
***V. labrusca* GVIT 1306**	*V. labrusca*	PI_588648	MF	M	4.76E+00	8.82E+31
***V. palmata* RU-66-8**	*V. palmata*	PI_588467	FF	F	9.09E-02	2.74E-32
***V. palmate* GV 25-3-20**	*V. palmate*	PI_588201	MF	M	1.30E+00	3.46E+21
***V. riparia* No. 37**	*V. riparia*	PI_588259	FF	F	9.09E-02	4.00E-29
***V. riparia* HP-1**	*V. riparia*	PI_588271	MF	M	1.33E+00	1.32E+21
***V. rupestris* 'Pillans'**	*V. rupestris*	PI_588174	MF	M	1.33E+00	1.65E+27
***V. rupestris* R-65-36**	*V. rupestris*	PI_588147	MF	M	3.20E+01	5.16E+37
***V. rupestris* B 38**	*V. rupestris*	PI_588160	FF	F	9.09E-02	5.38E-37
***V. sylvestris* DVIT_3597**	*V. vinifera* ssp. *sylvestris*	DVIT_3597	FF	F	5.00E-01	3.06E-48
***V. sylvestris* DVIT_3555**	*V. vinifera* ssp. *sylvestris*	DVIT_3555	FF	F	9.09E-02	1.75E-59

***V. sylvestris* DVIT_3615**	*V. vinifera* ssp. *sylvestris*	DVIT_3615	MF	M	1.33E+00	3.71E+45
***V. sylvestris* DVIT_3592**	*V. vinifera* ssp. *sylvestris*	DVIT_3592	MF	M	3.20E+01	3.45E+24
**Cabernet Franc**	*V. vinifera* ssp. *vinefera*	NA	HF	H	4.07E-44	2.15E+09
**Chardonnay**	*V. vinifera* ssp. *vinefera*	NA	HH	H	9.09E-02	9.24E+28
**Riesling**	*V. vinifera* ssp. *vinefera*	NA	HH	H	5.00E-01	6.72E+44
**Cabernet Sauvignon**	*V. vinifera* ssp. *vinefera*	NA	HF	H	9.09E-02	3.89E+35
**Pinot Gris**	*V. vinifera* ssp. *vinefera*	NA	HF	H	9.09E-02	1.56E+31
**Pinot Noir**	*V. vinifera* ssp. *vinefera*	NA	HF	H	5.00E-01	2.92E+35
**Sangiovese**	*V. vinifera* ssp. *vinefera*	NA	HF	H	2.14E-01	2.41E+77
**Vidal**	*V. vinifera* ssp. *vinefera*	NA	HF	H	9.09E-02	2.07E+38
**Merlot**	*V. vinifera* ssp. *vinefera*	NA	HF	H	9.09E-02	1.63E+38
**Traminette**	*V. vinifera* ssp. *vinefera*	NA	HF	H	9.09E-02	3.14E+86
**Valvin Muscat**	*V. vinifera* ssp. *vinefera*	NA	HF	H	9.09E-02	7.70E+81
***V. vulpina* Rem 15-77**	*V. vulpina*	PI_483180	FF	F	9.09E-02	4.14E-34
***V. vulpina* Rem 29-77**	*V. vulpina*	PI_483184	MF	M	4.76E+00	1.23E+36
***V. vulpina* Rem 38-77**	*V. vulpina*	PI_483188	FF	F	9.09E-02	4.05E-41
***V. vulpina* Rem 40-77**	*V. vulpina*	PI_483189	MF	M	4.76E+00	3.82E+43

Table S3 Cont. Bulked samples

Sample_name	Population	Samples in bulk	Haplotype	Phenotype
**Hor×ill547_HM**	Horizon(HH) × Ill547-1(MF)	361018;361025;361052;361079;363006	HM	M
**Hor×illi547_HF**	Horizon(HH) × Ill547-1(MF)	361022;363034;363067;363072;363089	HF	H
**Chardonnay×Cinerea_HM**	Chardonnay(HH)×V. CinereaB9(MF)	454064;454069;454075;454083;455032	HM	M
**Chardonnay×Cinerea_HF**	Chardonnay(HH)×V. CinereaB9(MF)	455033;455066;454052;454063;455015	HF	H
**Hor×cinerea_HM**	Horizon(HH) × V.CinereaB9(MF)	456011;456023;456101;457013;455110	HM	M
**Hor×cinerea_HF**	Horizon(HH) × V.CinereaB9(MF)	456048;457015;457019;457037;455105	HF	H

In female grapevine, Bayes Factor (BF) in A and C regions are less than 1. In male grapevine, Bayes Factor in A and C regions are both greater than 1. In the hermaphroditic grapevine, the Bayes Factor in the A region is less than 1 while in the C region is greater than 1. Cells with BF less than 1 are with red font. *Most likely a sampling error.

Table S4 Flower sex prediction and phenotype for rhAmpSeq samples

Sample name	USDA ID	Common name	Species	Observed phenotype	Predicted phenotype
**PI_588465**	PI_588465	89/82	*V. piasezkii*	5	5
**PI_597257**	PI_597257	90/82	*V. piasezkii*	5	5
**PI_588385**	PI_588385	GVIT_693	*V. amurensis*	1	1
**PI_588635**	PI_588635	GVIT_1277	*V. amurensis*	5	5
**PI_588637**	PI_588637	GVIT_1282	*V. amurensis*	1	1
**PI_588639**	PI_588639	GVIT_1285	*V. amurensis*	5	5
**PI_588641**	PI_588641	GVIT_1288	*V. amurensis*	1	1
**PI_588631**	PI_588631	GVIT_1267	*V. amurensis*	5	5
**PI_588633**	PI_588633	GVIT_1270	*V. amurensis*	5	5
**PI_588629**	PI_588629	GVIT_1264	*V. amurensis*	5	5
**PI_588630**	PI_588630	GVIT_1265	*V. amurensis*	5	5
**PI_588467**	PI_588467	RU-66-8	*V. spp.*	5	5
**PI_588155**	PI_588155	RU-66-10	*V. palmata*	1	1
**PI_588233**	PI_588233	Cache 8	*V. palmata*	5	5
**PI_588677__BIvDNAgut0034C10_B12**	PI_588677	JEG 3	*V. aestivalis*	4	5
**PI_588138**	PI_588138	GV 21-46-98	*V. labrusca*	1	1
**PI_483130**	PI_483130	Rem NE 4	*V. labrusca*	1	1
**PI_483137__BIvDNAgut0033C09_A02**	PI_483137	Rem 46-77	*V. aestivalis*	1	1
**PI_483138**	PI_483138	Rem 48-77	*V. aestivalis*	2	1
**PI_483151__BIvDNAgut0033C09_G12**	PI_483151	Rem NE 9	*V. labrusca*	5	5
**PI_588165**	PI_588165	Alba	*V. labrusca*	5	5
**PI_483133**	PI_483133	Rem NE 19	*V. labrusca*	1	1
**PI_483152**	PI_483152	Rem NE 11	*V. labrusca*	5	5
**PI_483150**	PI_483150	Rem NE 8	*V. labrusca*	5	5
**PI_483155**	PI_483155	Rem NE 15	*V. labrusca*	5	5
**PI_483153**	PI_483153	Rem NE 12	*V. labrusca*	1	1
**PI_483154**	PI_483154	Rem NE 13	*V. labrusca*	1	1
**PI_588194**	PI_588194	Dunkel 1	*V. labrusca*	5	5
**PI_588584**	PI_588584	Grem-5	*V. labrusca*	5	5
**PI_483148**	PI_483148	Rem 46-75	*V. labrusca*	5	5
**PI_483164__BIvDNAgut0032C08_H02**	PI_483164	Rem 47-77	*V. labrusca*	5	5
**PI_483145**	PI_483145	Rem 26-75	*V. labrusca*	5	5
**PI_588647__BIvDNAgut0034C10_D03**	PI_588647	GVIT_1304	*V. labrusca*	1	1

**PI_588585__BIvDNAgut0032C08_H10**	PI_588585	Wild Red 2	*V. labrusca*	5	5
**PI_588583**	PI_588583	Grem-4	*V. labrusca*	4	5
**PI_588648**	PI_588648	GVIT_1306	*V. labrusca*	1	1
**PI_483159**	PI_483159	Rem NE 24	*V. labrusca*	1	1
**PI_483161**	PI_483161	Rem NE 26	*V. labrusca*	1	1
**PI_483147**	PI_483147	Rem 43-75	*V. labrusca*	5	5
**PI_483149**	PI_483149	Rem 5-77	*V. labrusca*	1	1
**PI_483160**	PI_483160	Rem NE 25	*V. labrusca*	5	5
**PI_483146**	PI_483146	Rem 33-75	*V. labrusca*	1	1
**PI_483157**	PI_483157	Rem NE 20	*V. labrusca*	2	1
**PI_588626**	PI_588626	GVIT_1257	*V. aestivalis*	4	5
**PI_483185**	PI_483185	Rem 30-77	*V. aestivalis*	1	1
**PI_588540**	PI_588540	GBC 11	*V. rupestris*	2	1
**PI_588625**	PI_588625	B 65-20	*V. cinerea var. helleri*	1	1
**PI_588210__BIvDNAgut0039D03_B01**	PI_588210	B 65-7	*V. cinerea var. helleri*	5	5
**PI_588466**	PI_588466	Mazade	*V. cinerea var. helleri*	1	1
**PI_588205**	PI_588205	GV 21-46-75	*V. cinerea var. helleri*	5	5
**PI_588220__BIvDNAgut0031C07_E01**	PI_588220	B 55	*V. cinerea*	5	5
**PI_588575__BIvDNAgut0034C10_G07**	PI_588575	C-65-21	*V. cinerea*	5	5
**PI_588678__BIvDNAgut0034C10_H07**	PI_588678	JEG 2 SPR CR	*V. cinerea*	5	5
**PI_588685__BIvDNAgut0034C10_H11**	PI_588685	JEG 3 IGLOO	*V. cinerea*	5	5
**PI_588258__BIvDNAgut0030C06_H08**	PI_588258	No. 14	*V. riparia*	5	5
**PI_588352__BIvDNAgut0030C06_F08**	PI_588352	C-66-6	*V. cinerea*	1	1
**PI_588222**	PI_588222	C-66-7	*V. cinerea*	5	5
**PI_588199**	PI_588199	C-66-14	*V. cinerea*	1	1
**PI_588356**	PI_588356	GV 25-21-91	*V. cinerea*	1	1
**PI_588154**	PI_588154	B 9	*V. cinerea*	1	1
**PI_588143**	PI_588143	Ill 41	*V. cinerea*	5	5

**PI_588217__BIvDNAgut0031C07_G03**	PI_588217	B 27	*V. cinerea*	5	5
**PI_588398__BIvDNAgut0032C08_D04**	PI_588398	Barrett 1	*V. cinerea*	5	5
**PI_588186**	PI_588186	Ill 58	*V. cinerea*	5	5
**PI_588221__BIvDNAgut0031C07_H05**	PI_588221	B 58	*V. cinerea*	5	5
**PI_588218**	PI_588218	B 47	*V. cinerea*	5	5
**PI_588197**	PI_588197	Ill 49	*V. cinerea*	1	1
**PI_588372**	PI_588372	Ill 194-1	*V. cinerea*	1	1
**PI_588208**	PI_588208	Ill 65	*V. cinerea*	4	5
**PI_597232__BIvDNAgut0034C10_H08**	PI_597232	Ill 23	*V. cinerea*	5	5
**PI_588134**	PI_588134	Ill 45	*V. cinerea*	1	1
**PI_588328__BIvDNAgut0033C09_B12**	PI_588328	Ill 66	*V. cinerea*	5	5
**PI_588329**	PI_588329	Ill 24	*V. cinerea*	1	1
**PI_588174**	PI_588174	Pillans	*V. rupestris*	1	1
**PI_588198**	PI_588198	Ill 46	*V. cinerea*	1	1
**PI_588146**	PI_588146	R-66-4	*V. rupestris*	5	5
**PI_483176**	PI_483176	Tom's Favorite	*V. riparia*	5	5
**PI_483181**	PI_483181	Rem NE 22	*V. riparia*	5	5
**PI_588204**	PI_588204	GVIT_247	*V. riparia*	2	1
**PI_588190__BIvDNAgut0027C03_H02**	PI_588190	Pulliat	*V. riparia*	5	5
**PI_588395__BIvDNAgut0031C07_E02**	PI_588395	GVIT_704	*V. rupestris*	1	1
**PI_588415**	PI_588415	Constantia	*V. rupestris*	1	1
**PI_588574**	PI_588574	Wichita Refuge	*V. rupestris*	5	5
**PI_588231**	PI_588231	Alphonse de Serres	*V. rupestris*	5	5
**PI_588335**	PI_588335	GV 25-8-114	*V. rupestris*	2	1
**PI_588223**	PI_588223	R-65-43	*V. rupestris*	1	1
**PI_588225**	PI_588225	R-65-47	*V. rupestris*	5	5
**PI_588228**	PI_588228	R-66-15	*V. rupestris*	2	1
**PI_588355**	PI_588355	R-66-2	*V. rupestris*	5	5
**PI_588401**	PI_588401	RU-66-2	*V. spp.*	5	5
**PI_588224**	PI_588224	R-65-44	*V. rupestris*	5	5
**PI_588229**	PI_588229	R-66-24	*V. rupestris*	2	1

**PI_588147**	PI_588147	R-65-36	*V. rupestris*	1	1
**PI_588181**	PI_588181	R-66-3	*V. rupestris*	1	1
**PI_588230**	PI_588230	R-66-31	*V. rupestris*	2	1
**PI_588330**	PI_588330	R-67-3	*V. rupestris*	2	1
**PI_588333__BIvDNAgut0030C06_E08**	PI_588333	R-67-2	*V. rupestris*	1	1
**PI_483173**	PI_483173	Rem 82-76	*V. riparia*	5	5
**PI_588399__BIvDNAgut0031C07_F02**	PI_588399	150-44	*V. spp.*	5	5
**PI_588324__BIvDNAgut0031C07_B02**	PI_588324	GV 24-10-169	*V. acerifolia*	5	5
**PI_588325__BIvDNAgut0031C07_H02**	PI_588325	GV 24-10-165	*V. acerifolia*	5	5
**PI_483165**	PI_483165	Rem 55-75	*V. riparia*	5	5
**PI_588378**	PI_588378	DVIT 1148	*V. acerifolia*	5	5
**PI_483178**	PI_483178	Rem 4-77	*V. riparia*	1	1
**PI_588201**	PI_588201	GV 25-3-20	*V. palmata*	1	1
**PI_588442**	PI_588442	GVIT_780	*V. acerifolia*	1	1
**PI_588483**	PI_588483	Rem NE 21	*V. riparia*	5	5
**PI_588646**	PI_588646	GVIT_1300	*V. acerifolia*	2	1
**PI_318684**	PI_318684	25-1-64	*V. acerifolia*	5	5
**PI_588393**	PI_588393	GVIT_702	*V. acerifolia*	1	1
**PI_588440**	PI_588440	Site 14	*V. riparia*	1	1
**PI_483182**	PI_483182	Rem 24-77	*V. riparia*	1	1
**PI_588331**	PI_588331	St. George	*V. rupestris*	1	1
**PI_588565**	PI_588565	Grem	*V. riparia*	5	5
**PI_588568**	PI_588568	Crosby rootstock	*V. riparia*	5	5
**PI_588456**	PI_588456	GVIT_823	*V. riparia*	5	5
**PI_588404**	PI_588404	Wichita	*V. riparia*	1	1
**PI_588400__BIvDNAgut0031C07_D02**	PI_588400	GVIT_710	*V. riparia*	5	5
**PI_588435**	PI_588435	2F	*V. riparia*	5	5
**PI_588437**	PI_588437	1F	*V. riparia*	5	5
**PI_588439**	PI_588439	GVIT_775	*V. riparia*	2	1
**PI_588054**	PI_588054	Iowa 7	*V. riparia*	5	5
**PI_588711**	PI_588711	Bougher	*V. riparia*	5	5
**PI_483166**	PI_483166	Rem 65-76	*V. riparia*	5	5
**PI_483170**	PI_483170	Rem 73-76	*V. riparia*	5	5
**PI_483171**	PI_483171	Rem 77-76	*V. riparia*	5	5
**PI_483169**	PI_483169	Rem 70-76	*V. riparia*	2	1

**PI_483172**	PI_483172	Rem 81-76	*V. riparia*	4	5
**PI_483174**	PI_483174	Rem 83-76	*V. riparia*	5	5
**PI_255189**	PI_255189	Urbana 1	*V. riparia*	5	5
**PI_588345**	PI_588345	Quebec	*V. riparia*	5	5
**PI_483167**	PI_483167	Rem 66-76	*V. riparia*	2	1
**PI_483168**	PI_483168	Rem 67-75	*V. riparia*	2	1
**PI_313922**	PI_313922	Tarnau	*V. riparia*	2	1
**PI_588718__BIvDNAgut0035C11_A01**	PI_588718	L505	*V. riparia*	5	5
**PI_588653**	PI_588653	Okoboji	*V. riparia*	5	5
**PI_588457**	PI_588457	Illinois 7	*V. riparia*	1	1
**PI_588259**	PI_588259	No. 37	*V. riparia*	5	5
**PI_588260**	PI_588260	No. 64	*V. riparia*	5	5
**PI_588354**	PI_588354	B 75	*V. riparia*	1	1
**PI_588214**	PI_588214	Gloire de Montpellier	*V. riparia*	1	1
**PI_588369**	PI_588369	Dyson	*V. riparia*	1	1
**PI_588261**	PI_588261	No. 74	*V. riparia*	5	5
**PI_588262**	PI_588262	No. 89	*V. riparia*	5	5
**PI_588455**	PI_588455	GVIT_820	*V. riparia*	5	5
**PI_588347**	PI_588347	B 50	*V. riparia*	5	5
**PI_588562**	PI_588562	Wisconsin 2	*V. riparia*	5	5
**PI_588406**	PI_588406	Meissner 13	*V. riparia*	5	5
**PI_588438**	PI_588438	GVIT_774	*V. riparia*	5	5
**PI_279897**	PI_279897	Grand Glabre	*V. riparia*	1	1
**PI_588304**	PI_588304	GV 21-46-23	*V. riparia*	5	5
**PI_588271**	PI_588271	HP-1	*V. riparia*	1	1
**PI_588275**	PI_588275	HP-2	*V. riparia*	5	5
**PI_588346**	PI_588346	RA-66-12	*V. riparia*	2	1
**PI_588274**	PI_588274	62-11-42	*V. riparia*	5	5
**PI_588510**	PI_588510	RA-66-8	*V. riparia*	5	5
**PI_588349**	PI_588349	RA-66-10	*V. riparia*	1	1
**PI_588374**	PI_588374	RA-66-11	*V. riparia*	1	1
**PI_588272**	PI_588272	62-9-39	*V. riparia*	2	1
**PI_588373**	PI_588373	RA-66-6	*V. riparia*	1	1
**PI_588273**	PI_588273	62-7-2	*V. riparia*	2	1
**PI_588276**	PI_588276	62-8-138	*V. riparia*	5	5
**PI_588344**	PI_588344_2	RA-66-3	*V. riparia*	2	1
**PI_588350**	PI_588350	RA-66-5	*V. riparia*	1	1
**PI_588269**	PI_588269	62-8-160	*V. riparia*	5	5
**PI_588270**	PI_588270	62-9-44	*V. riparia*	5	5
**PI_588353**	PI_588353	RA-66-7	*V. riparia*	5	5

Flower sex scale:1=Male; 2=Male to Hermaphrodite; 3=Hermaphrodite; 4=Female, with upright stamina 5=Female.

Dataset S1. Accessions characters, population assignment and flower sex prediction of shotgun sequenced wild and cultivated grapevines used in this study. In female grapevine, Bayes Factor (BF) in A and C regions are less than 1. In male grapevine, Bayes Factor in A and C regions are both greater than 1. In the hermaphroditic grapevine, the Bayes Factor in the A region is less than 1 while in the C region is greater than 1. Cells with BF larger than 1 are filled with light blue, while cells with BF less than 1 are filled with light red.

Dataset S2. The genomic position of 1,066 single nucleotide polymorphisms that co-segregated with flower sex phenotype.

Dataset S3. A pedigree network of the sampled cultivars with predicted haplotype

Dataset S4. Markers we have designed and test at the Ampseq and rhAmpSeq platform

SI References

1. C. Zou, *et al.*, Haplotyping the *Vitis* collinear core genome with rhAmpSeq improves marker transferability in a diverse genus. *Nature communications* **11**, 413 (2020).

2. S. Patel, *et al.*, Draft genome of the Native American cold hardy grapevine *Vitis riparia* Michx. "Manitoba 37." *Horticulture research* **7**, 1–13 (2020).

3. M. Massonnet, *et al.*, The genetic basis of sex determination in grapes. *Nature communications* **11**, 1–12 (2020).

4. A. Minio, M. Massonnet, R. Figueroa-Balderas, A. Castro, D. Cantu, Diploid Genome Assembly of the Wine Grape Carménère. *G3: Genes, Genomes, Genetics* **9**, 1331–1337 (2019).

5. S. Ou, *et al.*, Effect of sequence depth and length in long-read assembly of the maize inbred NC358. *Nature Communications* **11**, 2288 (2020).

6. S. Deschamps, *et al.*, A chromosome-scale assembly of the sorghum genome using nanopore sequencing and optical mapping. *Nature Communications* **9**, 4844 (2018).

7. A. Minio, *et al.*, Iso-Seq Allows Genome-Independent Transcriptome Profiling of Grape Berry Development. *G3: Genes, Genomes, Genetics* **9**, 755–767 (2019).

8. H. Li, Minimap2: pairwise alignment for nucleotide sequences. *Bioinformatics* **34**, 3094–3100 (2018).

9. A. Smit, R. Hubley, P. Green, *RepeatMasker Open-4.0. 2013–2015* (2015).

10. F. Hahne, R. Ivanek, Visualizing Genomic Data Using Gviz and Bioconductor. *Methods Mol Biol* **1418**, 335–351 (2016).

11. H. Li, R. Durbin, Fast and accurate short read alignment with Burrows-Wheeler transform. *Bioinformatics (Oxford, England)* **25**, 1754–1760 (2009).

12. Y. Zhou, M. Massonnet, J. S. Sanjak, D. Cantu, B. S. Gaut, Evolutionary genomics of grape (*Vitis vinifera* ssp. *vinifera*) domestication. *Proceedings of the National Academy of Sciences of the United States of America* **114**, 11715–11720 (2017).

13. K. I. Kendig, *et al.*, Sentieon DNASeq Variant Calling Workflow Demonstrates Strong Computational Performance and Accuracy. *Frontiers in genetics* **10**, 736 (2019).

14. R. Poplin, *et al.*, Scaling accurate genetic variant discovery to tens of thousands of samples. *bioRxiv* (2018) https:/doi.org/10.1101/201178.

15. S. Myles, *et al.*, Genetic structure and domestication history of the grape. *Proc. Natl. Acad. Sci. U.S.A.* **108**, 3530–3535 (2011).

16. A. Di Genova, *et al.*, Whole genome comparison between table and wine grapes reveals a comprehensive catalog of structural variants. *BMC plant biology* **14**, 7 (2014).

17. Y. Zhou, *et al.*, The population genetics of structural variants in grapevine domestication. *Nat Plants* **5**, 965–979 (2019).

18. R. Das, R. Roy, N. Venkatesh, Using Ancestry Informative Markers (AIMs) to Detect Fine Structures Within Gorilla Populations. *Frontiers in genetics* **10**, 43 (2019).

19. S. Purcell, *et al.*, PLINK: a tool set for whole-genome association and population-based linkage analyses. *American journal of human genetics* **81**, 559–575 (2007).

20. A. Raj, M. Stephens, J. K. Pritchard, fastSTRUCTURE: variational inference of population structure in large SNP data sets. *Genetics* **197**, 573–589 (2014).

21. A. Antcliff, Inheritance of sex in *Vitis*. *Annales de l'Amelioration des Plantes* **30**, 113–122 (1980).

22. J. Battilana, *et al.*, Linkage mapping and molecular diversity at the flower sex locus in wild and cultivated grapevine reveal a prominent SSR haplotype in hermaphrodite plants. *Molecular biotechnology* **54**, 1031–1037 (2013).

23. S. Angiuoli, S. Salzberg, Mugsy: fast multiple alignment of closely related whole genomes. *Bioinformatics* **27**, 334–42 (2011).

24. J. Castresana, Selection of conserved blocks from multiple alignments for their use in phylogenetic analysis. *Molecular biology and evolution* **17**, 540–552 (2000).

25. A. Löytynoja, Phylogeny-aware alignment with PRANK. *Methods Mol Biol* **1079**, 155–170 (2014).

26. D. Darriba, G. L. Taboada, R. Doallo, D. Posada, jModelTest 2: more models, new heuristics and parallel computing. *Nature methods* **9**, 772 (2012).

27. A. Stamatakis, RAxML version 8: a tool for phylogenetic analysis and post-analysis of large phylogenies. *Bioinformatics (Oxford, England)* **30**, 1312–1313 (2014).

28. A. J. Drummond, M. A. Suchard, D. Xie, A. Rambaut, Bayesian phylogenetics with BEAUti and the BEAST 1.7. *Molecular biology and evolution* **29**, 1969–1973 (2012).

29. Z.-Y. Ma, *et al.*, Phylogenomics, biogeography, and adaptive radiation of grapes. *Molecular phylogenetics and evolution* **129**, 258–267 (2018).

30. G. Gremme, S. Steinbiss, S. Kurtz, GenomeTools: a comprehensive software library for efficient processing of structured genome annotations. *IEEE/ACM transactions on computational biology and bioinformatics* **10**, 645–656 (2013).

31. R. Edgar, MUSCLE: multiple sequence alignment with high accuracy and high throughput. *Nucleic Acids Res* **32**, 1792–1797 (2004).

32. M. Kimura, A simple method for estimating evolutionary rates of base substitutions through comparative studies of nucleotide sequences. *Journal of molecular evolution* **16**, 111–120 (1980).

33. G. Csardi, T. Nepusz, The igraph software package for complex network research. *InterJournal, complex systems* **1695**, 1–9 (2006).

34. P. Bradbury, *et al.*, TASSEL: software for association mapping of complex traits in diverse samples. *Bioinformatics* **23**, 2633–2635 (2007).

35. A. Dobin, *et al.*, STAR: ultrafast universal RNA-seq aligner. *Bioinformatics (Oxford, England)* **29**, 15–21 (2013).

36. T. Koressaar, M. Remm, Enhancements and modifications of primer design program Primer3. *Bioinformatics (Oxford, England)* **23**, 1289–1291 (2007).

37. S. Yang, *et al.*, A next-generation marker genotyping platform (AmpSeq) in heterozygous crops: a case study for marker-assisted selection in grapevine. *Horticulture research* **3**, 16002 (2016).

38. H. Li, A statistical framework for SNP calling, mutation discovery, association mapping and population genetical parameter estimation from sequencing data. *Bioinformatics (Oxford, England)* **27**, 2987–2993 (2011).
